# Fluorimetric and CD Recognition between Various ds-DNA/RNA Depends on a Cyanine Connectivity in Cyanine-guanidiniocarbonyl-pyrrole Conjugate

**DOI:** 10.3390/molecules25194470

**Published:** 2020-09-29

**Authors:** Tamara Šmidlehner, Marta Košćak, Ksenija Božinović, Dragomira Majhen, Carsten Schmuck, Ivo Piantanida

**Affiliations:** 1 Division of Organic Chemistry and Biochemistry, Ruđer Bošković Institute, Bijenička Cesta 54, 10000 Zagreb, Croatia; tamara.smidlehner@ki.si (T.Š.); marta.koscak@irb.hr (M.K.); 2 Division of Molecular Biology, Ruđer Bošković Institute, Bijenička cesta 54, 10000 Zagreb, Croatia; ksenija.bozinovic@irb.hr (K.B.); dragomira.majhen@irb.hr (D.M.); 3 Institute of Organic Chemistry, University of Duisburg-Essen, 45141 Essen, Germany; carsten.schmuck@uni-due.de

**Keywords:** cyanine dyes, guanidiniocarbonyl-pyrrole, ds-DNA/RNA sensing, fluorescence, circular dichroism, mitochondria

## Abstract

Two novel isosteric conjugates of guanidiniocarbonyl-pyrrole and 6-bromo-TO (thiazole orange) were prepared, differing only in linker connectivity to cyanine (benzothiazole nitrogen vs. quinoline nitrogen). The quinoline analog was significantly more susceptible to aggregation in an aqueous medium, which resulted in induced circular dichroism (ICD; λ = 450–550 nm) recognition between A-T(U) and G-C basepair containing polynucleotides. The benzothiazole-isostere showed pronounced (four-fold) fluorimetric selectivity toward ds-RNA in comparison to any ds-DNA, at variance to its quinoline-analogue fluorescence being weakly selective to GC-DNA. Preliminary screening on human tumor and normal lung cell lines showed that both dyes very efficiently enter living cells and accumulate in mitochondria, causing moderate cytotoxic effects, and thus could be considered as lead compounds toward novel theragnostic mitochondrial dyes.

## 1. Introduction

Versatile applications of small molecule fluorescent probes targeting DNA/RNA in biochemical and biomedicinal applications have attracted enormous interest and, due to the versatility of applications, have become unavoidable tools for monitoring biological processes [1]. The research on DNA/RNA dyes has been mostly focused on the impact on the DNA/RNA function or selective/specific DNA/RNA labeling [2,3,4]. However, the huge complexity of DNA-coded processes, which do not depend only on coding DNA basepair sequences, but also include epigenetics, has only recently attracted attention [5,6].

Cyanine analogs are likely the most extensively used family of fluorogenic dyes, their particular advantage being non-emissive in free solution but strong emission fluorescence upon binding to the target [7,8,9]. On the other hand, our systematic work on aryl-guanidiniocarbonyl-pyrrole (GCP) derivatives characterized GCP moiety as a very useful building block in the design of new small molecules targeting DNA/RNA, particularly when conjugated with large aromatic fluorophores [10,11,12]. For instance, pyrene-GCP analogs have been shown to be very efficient single-molecule- multipurpose probes, being able to recognize between various ds-DNA and ds-RNA by simultaneous specific responses in fluorescence and circular dichroism [13,14]. Moreover, recently reported pyrene-GCP analog fluorimetric recognition of DNA/RNA could be reversibly controlled by pH [15].

We recently showed that isosteric cyanine conjugates with pyrene show some different properties in interaction with DNA/RNA and also proteins (BSA) as a result of different connectivity of the linker to the cyanine chromophore [16]. Very recently, we also prepared the first cyanine-GCP conjugate, which revealed promising selective fluorimetric and circular dichroism sensing between various ds-DNA or ds-RNA polynucleotides, which was additionally controllable by pH due to GCP [17]. Led by the aforementioned promising results, we designed and prepared novel analogs of cyanine-GCP conjugates, conjugates **1** and **2**, characterization of which we presented in this work.

**Scheme 1:**
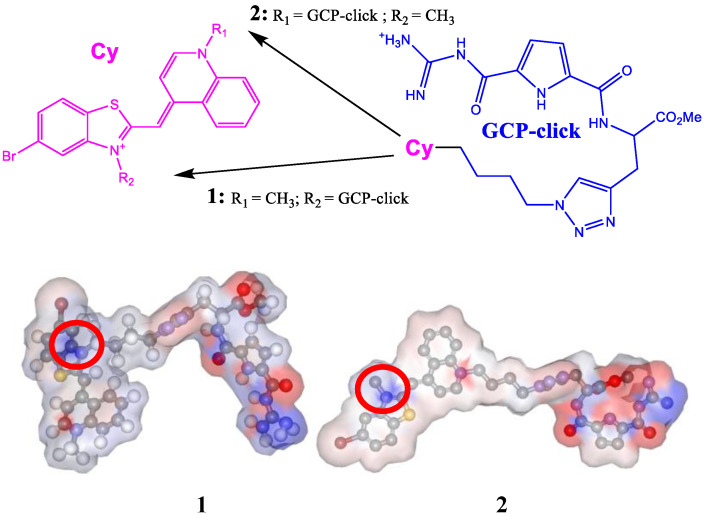
TOP: General structure of studied compounds **1** and **2**; DOWN: ball-and-stick models with superimposed van der Waals (VdW) radius surface denoting atom charge distribution (red-negative; blue positive), stressing the difference in the exposure of positive charge (red circles).

In the design of new conjugates (Scheme 1), we introduced an elongated linker with one more CH_2_^−^ group, with respect to the afore-studied analog [17] to increase flexibility, and we changed cyanine substituent “Cl” with “Br”. A larger volume of “Br” is likely to block intercalation of cyanine moiety between DNA/RNA base pairs, thus directing small molecule to the DNA or RNA grooves, which could result in the high sensitivity to binding site properties (namely, grooves of various ds-DNA or ds-RNA differ considerably—see Table S1, Supplementary Materials). Conjugate **1** was previously tested for inhibitor properties of dipeptidyl peptidase III (DPP III) [18].

The conjugates **1** and **2** are actually isosteres, differing in a reversed connectivity to cyanine chromophore (benzothiazole vs. quinoline), which resulted in more (**1**) or less (**2**) shielded position of permanent positive charge in the molecule (Scheme 1, bottom). Moreover, both molecules also have protonation-site (GCP), which tends to insert into DNA/RNA grooves only at weakly acidic conditions, thus allowing further control of DNA/RNA interaction by outer stimuli (pH change between 5 and 7). Since DNA/RNA are polyanions, and electrostatic interactions with small molecule cations usually contribute significantly to complex formation, it is to be expected that although isosteres, **1** and **2** would show significantly different patterns in DNA/RNA recognition, allowing exploration of the importance of steric, electrostatic and H-bonding impacts on DNA/RNA recognition.

**Scheme 2:**
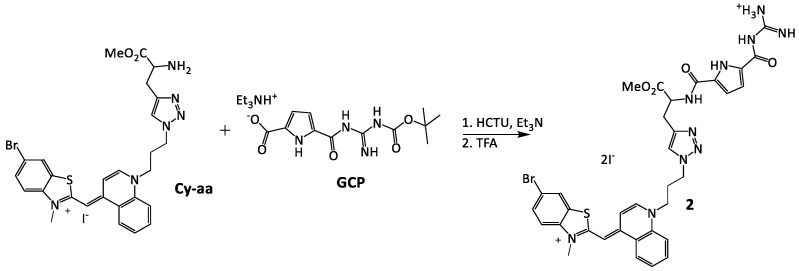
Synthetic route for the preparation of **2**, analogous to previous preparation of **1** [18].

Biorelevant applications of novel dyes are often related to their interactivity with living cells. The series of analogs of parent cyanine-amino acid (Cy-aa, Scheme 2) for the here prepared **1** and **2** already showed very efficient uptake in human cells and selective accumulation in mitochondria, accompanied by moderate cytotoxicity against human tumor cell lines (IC50 10–90 μM) [19]. The here prepared **1** and **2** are actually upgraded by the addition of a GCP unit, which itself is non-toxic and also known for efficiently entering cells, as well as acting as a transfection agent [20,21]. Thus, for novel Cy-amino acid-GCP conjugates **1** and **2,** questions remained whether they will retain efficient cellular uptake and, more important, whether mitochondrial selectivity of cyanine-amino acid part will also bring GCP unit to mitochondria. If so, GCP as a very rich H-bonding moiety and being pH-sensitive could influence the bioactivity of new conjugates. 

## 2. Results and Discussion

### 2.1. Chemistry

The **2** was prepared as shown on Scheme 2, starting from the corresponding cyanine-amino acids (**Cy-aa**) [19] and guanidiniocarbonyl-pyrrole (**GCP**) [10,11,12], following a similar procedure used previously for the preparation of its analog **1** [18]. Namely, the reaction mixture of Cy-amino acid and GCP in acetonitrile with O-(1H-6-Chlorobenzotriazole-1-yl)-1,1,3,3-tetramethyluronium hexafluorophosphate (HCTU) and Et_3_N was left stirring at room temperature under argon for three days. Boc-protected product was deprotected by the addition of TFA, the solvent was evaporated, and the crude product was recrystallized from the methanol-ether mixture and washed with water to give a pure product in 60% yield as a red solid.

### 2.2. Characterization of 1 and 2 in Aqueous Solutions

**Figure 1:**
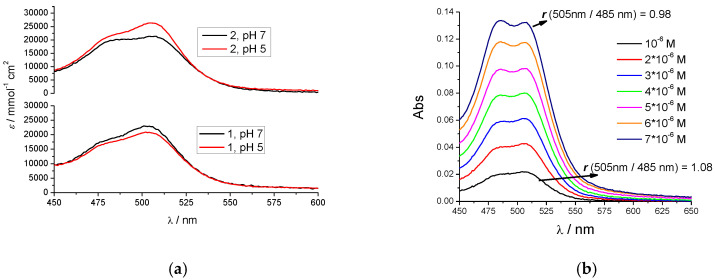
(**a**) The UV-Vis spectra of **1** and **2** (*c* = 2 × 10^−6^ M) at pH 7.0 and pH 5.0 (sodium cacodylate buffer, *I* = 0.05 M). Note the difference in ratio ***r*** (505 nm/485 nm) at different pH only for **2** but not for **1**; (**b**) The absorbance dependence on *c*(**2**); note the concentration-dependent change of ratio ***r***(505 nm/485 nm).

Both compounds were well-soluble in water, allowing preparation of stock solutions at mM concentrations, stable over longer periods stored in a refrigerator. The UV/Vis spectra of **1** and **2** were collected at pH 7.0 and pH 5.0 to monitor both the neutral and protonated form of GCP. At both pH (Figure 1) **1** and **2** revealed two pronounced absorption regions, one (λ_max_ ≈ 300 nm) originating from overlapped absorbancies of guanidiniocarbonyl-pyrrole (GCP) and triazole moieties and another absorption band in the visible range attributed to cyanine dye (λ_max_ = 480–510 nm). For both compounds at micromolar concentrations, absorbancies were proportional to the concentration (Supplementary Materials), allowing determination of molar extinction coefficients (Supplementary Materials in Table S2).

Closer analysis of UV/Vis data revealed distinct differences between **1** and **2**, which could be correlated to the connectivity of cyanine to the tether. Namely, the benzothiazole-tethered **1** UV/Vis spectrum has a dominant maximum at λ = 505 nm, the constant ratio ***r*** (505 nm/485 nm) was retained over the entire span of micromolar concentrations (Supplementary Materials), and also this ratio did not change with pH (Figure 1a), nor it changed with the temperature increase (Supplementary Materials in Figure S5, LEFT); all these properties typical for non-aggregated cyanines [9,22]. Contrary to **1**, the UV/Vis spectrum of quinoline-tethered **2** changed within the micromolar concentrations (Figure 1b), whereby a decrease of the ratio ***r*** (505 nm/485 nm) could be attributed to the increasing H-type cyanine aggregation, also supported by equivalent changes upon temperature increase (Supplementary Materials in Figure S5, RIGHT) [9,22].

Moreover, the for **2,** the pH-dependent difference in ratio ***r*** (505 nm/485 nm) (Figure 1a) clearly suggested more efficient aggregation at neutral conditions in comparison to pH 5, likely because the repulsive interactions between the protonated guanidiniocarbonyl-pyrrole (GCP) moieties hamper the efficient aromatic stacking (necessary for aggregation).

Previously studied **Cy-GCP** revealed aggregation properties in UV/Vis spectrum [17] comparable to its close analog **2**, pointing out that quinoline-tethered cyanines conjugated with GCP moiety are more prone to aggregation in comparison to benzothiazole-analogs (**1**). 

Both **1** and **2** have negligible fluorescence emission due to well-known non-emissive relaxation of cyanines in a free state [9] and, although both compounds are chiral, their CD spectra are very weak at studied conditions (data not shown) due to the distance between chiral center and chromophore.

### 2.3. Interactions with ds-DNA, ds-RNA, and ss-RNA

The choice of ds-DNA and ds-RNA in this study was driven by significant differences in the secondary structure of particular homogeneous polynucleotide sequences (for detailed structural features, see Supplementary Materials in Table S1): poly dAdT-poly dAdT are characterized by typical B-helical structure and minor groove ideal for small molecule binding; poly dGdC-poly dGdC minor groove is sterically blocked by guanine amino groups significantly hampering small molecule insertion; poly A—poly U presenting typical RNA A-helical structure with a major groove as a potential target for small molecules. Also, for comparison reasons, we used naturally isolated DNA from *calf thymus* (ct-DNA), characterized by a typical B-helical structure and an equal amount of AT- and GC-base pairs. All chosen polynucleotides are 100–200 base pairs long, ensuring a large quantity of identical, mutually independent binding sites for a small molecule and thus making the contribution of molecule binding at polynucleotide termini negligible.

#### 2.3.1. Thermal Denaturation Experiments

The thermal denaturation experiments provide information about the ds- polynucleotide helix thermal stability as a function of interaction with added small molecules [23]. The difference between the *Tm* value of free ds-polynucleotide and a complex with a small molecule (Δ*Tm* value) is an important factor in the characterization of small molecule/ds-polynucleotide interactions. For instance, moderate to strong stabilization (Δ*Tm* > 5 °C) supports pronounced intercalative or minor groove binding interaction [24], whereas weak or negligible stabilization (Δ*Tm* = 0–5 °C) suggests a binding process driven mostly by hydrophobic effect accompanied with weak H-bonding and/or electrostatic interactions—usually excluding classical intercalation as a binding mode.

The impact of **1** and **2** conjugates on stabilization of ds-polynucleotides at neutral conditions (pH 7) was rather weak and similar for all studied DNA/RNA (Table 1), excluding classical intercalation as a dominant binding mode. However, for both compounds the stabilization effect increased considerably at pH 5, which can be attributed to the protonation of **GCP** and its additional electrostatic interactions with the negatively charged DNA/RNA backbone. Intriguingly, acidic pH had a less pronounced effect on AT-DNA stabilization, likely due to the deep insertion of GCP into a convenient minor groove [25,26].

#### 2.3.2. Fluorimetric Titrations

The tendency of one studied dye to aggregate at *c* > 2 µM hampered accurate titrations in UV/Vis medium. However, the well-known property of cyanine dyes to switch-on strong fluorescence upon DNA/RNA binding allowed fluorimetric titration at non-aggregative conditions. 

**Figure 2:**
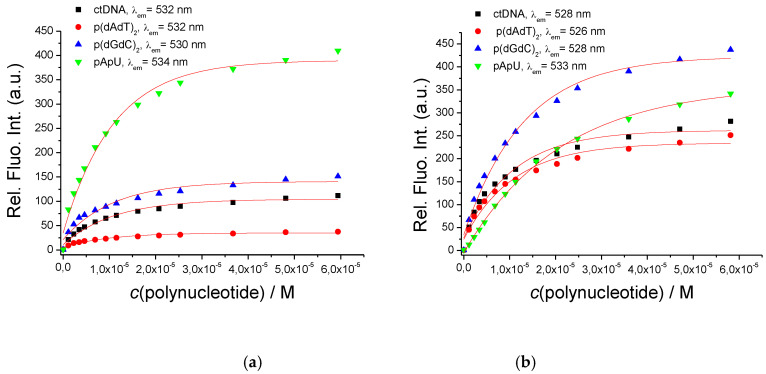
Changes in fluorescence of: (**a**) **1** (λ_exc_ = 505 nm, *c* = 5 × 10^−7^ M) or (**b**) **2** (λ_exc_ = 505 nm, *c* = 5 × 10^−7^ M); upon addition of polynucleotides. Done at pH 7.0; sodium cacodylate buffer, *I* = 0.05 M.

Indeed, buffered solutions of **1** and **2**, intrinsically non-emissive, exhibited strong fluorescence upon the addition of any ds-DNA or ds-RNA. However, the intensity of emission was strongly dependent on a compound structure as well as on the secondary structure of particular ds-polynucleotide (Figure 2). Conjugate **1** showed strong, four-fold emission preference toward ds-RNA, at variance to its isostere **2** being generally less selective, but emitting the most brightly for GC-DNA. Both dyes reveal the weakest emission for AT-DNA. Such different responses suggest that both dyes bind in a similar manner to AT-DNA, whereas their binding to GC-DNA or AU-RNA significantly differs as a consequence of a fine interplay between dye structure and peculiar properties of DNA vs. RNA binding sites. Namely, only AT-DNA has a minor groove convenient for small molecule binding, in which both **1** and **2** bind similarly, giving a similar response. The AU-RNA major groove (common binding site for small molecules [4]) is of similar width as-AT-DNA, but much deeper (Table S1)—obviously allowing the difference in binding of cyanine-chromophore of **1** in respect to **2**; while GC-DNA minor groove is sterically hindered by protruding amino groups of guanine which significantly limit the accommodation of small molecules, again being sensitive in structure difference between **1** and **2**.

We performed fluorimetric titrations also at pH 5, at which the GCP unit is protonated and, with an additional positive charge, could interact with negatively charged DNA/RNA backbone, increasing overall affinity (Supplementary Materials in Figure S24). Intriguingly, the fluorimetric response of **1** was identical to experiments done at pH 7, while **2** showed a slightly different fluorescence recognition pattern at pH 5, showing a small fluorimetric preference for mixed ct-DNA, followed by GC-DNA and AU-RNA. The reason why both new dyes (**1**, **2**) did not show a pronounced impact of pH on their fluorimetric response, as previously noted for e.g., series of pyrene—GCP analogs [13,14,15], is probably because cyanine fluorophore is cationic itself, and thus not so dependent in binding to DNA/RNA on the additional positive charge of GCP present only at pH 5.

All titration data were processed by non-linear fitting to the Scatchard equation (McGhee, von Hippel formalism) [28,29], giving binding constants (Table 2). Affinities of both dyes to all ds-polynucleotides were similar, thus suggesting that the observed difference in emission response is not due to the strength of interactions but more likely the result of fine differences in positioning of cyanine within the binding site and consequence freedom of rotation around methine bond. Further, somewhat stronger binding at pH 5 in comparison to pH 7 agrees nicely to the aforementioned stronger stabilization effects (Table 1) and could be attributed to the protonation of GCP moiety at weakly acidic conditions, yielding additional binding interaction.

#### 2.3.3. Circular Dichroism (CD) Experiments

To study in more structural detail the properties of complexes formed, we used circular dichroism (CD) spectrophotometry [30]. In addition, the small molecule could, upon binding to polynucleotides, acquire an induced (I)CD signal, positioned at the absorption bands of the small molecule, in this case in the range 300–550 nm, not coinciding with the DNA/RNA CD signals. The character and the magnitude of the ICD signal offer valuable information for the determination of binding modes (intercalation, agglomeration, groove binding, etc.) [31]. Minor groove binding to ds-DNA orientates the ligand approximately at 45° with respect to the DNA chiral axis, giving a strong positive ICD band. Intercalation brings the aromatic moiety of the ligand in a coplanar arrangement with the base pairs, giving only a weak ICD band (in the majority of cases with a negative sign due to parallel orientation of the transition vector of the ligand and the longer axis of the surrounding base pairs) [31,32].

The studied compounds are chiral, but the intensities of their CD spectra in the 230–500 nm range are negligible with respect to CD spectra of polynucleotides, allowing accurate correction of CD titrations.

**Figure 3:**
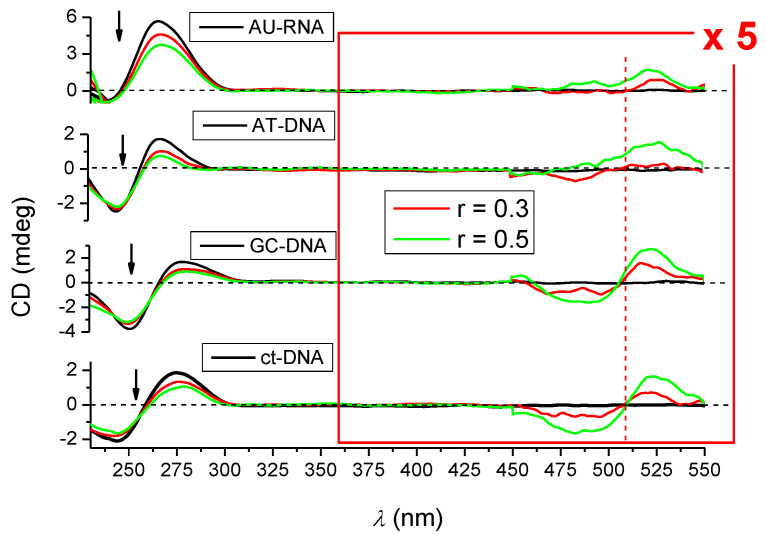
Circular dichroism (CD) titration of ds-DNAs and ds-RNA (▬; *c* = 2 × 10^−5^ M) with **2** at molar ratios ***r***_[2]/[polynucleotide]_ = 0.3 (▬) and 0.5 (▬). Note that marked range 450–550 nm was multiplied by 5 for better visibility. Done at pH 7, sodium cacodylate buffer, *I* = 0.05 M.

The addition of studied dyes resulted in a general decrease of DNA/RNA CD bands (240–290 nm, Supplementary Materials in Figures S25–S28), somewhat more pronounced for **2** in comparison with **1**. Such a decrease of DNA/RNA chirality is usually associated with the small molecule-induced unwinding of polynucleotide double helix [31,32]. Intriguingly, **1** induced by far the strongest decrease in the AU-RNA CD spectrum (Supplementary Materials in Figure S26d), which could be correlated to the strongest fluorescence emission increase of **1** upon addition of AU-RNA (Figure 2). On the other hand, **2** induced similar changes in CD spectra of all DNA/RNA (Figure 3) in accordance with similar emission increase (Figure 2).

At variance to all previously studied aryl-GCP conjugates [10,11,12,13,14,15] and even the Cy-GCP [17] (very close analog of **2**), no ICD bands of GCP chromophore (at λ = 300 nm) were observed, pointing out that for both, **1** and **2**, GCP moiety was not uniformly oriented in respect to the polynucleotide chiral axis [32], similar as previously noted for nucleobase-GCP analogs [33].

Detailed analysis of λ = 450–600 nm range (Figure 3, Supplementary Materials, S27) revealed very weak or negligible ICD bands for **1** at both pH, at variance with the much more pronounced ICD bands for **2** (Figure 3), the latter correlating with strong ICD bands of cyanine chromophore, as also seen previously for its close analog **Cy-GCP** [17]. Since all compounds (**1**, **2,** and **Cy-GCP [17]**) bind to ds-DNA/RNA with similar affinity (Table 2), it seems that quinoline-tethered cyanines (**2** and **Cy-GCP [17]**) attain more uniform orientation in respect to the polynucleotide chiral axis (thus yielding well-defined ICD bands) [32] in comparison to poorly oriented cyanine of benzothiazole-tethered analog **1**.

The ICD bands of **2** were exclusively positive for AT-DNA and AU-RNA, which is typical for single-molecule minor/major groove binding [31,32], at variance to bisignate ICD bands observed only for GC-containing DNAs, which are characteristic for groove aggregation/dimerization of cyanine dyes [31,32]. These bisignate bands can be correlated to the propensity of **2** for aggregation (see Figure 1), and likely to occur within DNA major groove, since GC-DNA minor groove is sterically blocked by alternatively protruding amino groups of guanine [25,26].

#### 2.3.4. Biological Evaluation of 1 and 2 on Human Cell Lines

The aim of the biological experiments performed was to verify the actual capability of these DNA/RNA binders to penetrate into cells, to visualize their intracellular location and subcellular targets, and evaluate their anti-proliferative effect, to identify their further potential applications as either lead compounds towards fluorescent anti-proliferative drugs, or as non-cytotoxic dyes suitable for intracellular applications or in biochemical studies on DNA and RNA [1,2,5,6].

**Figure 4:**
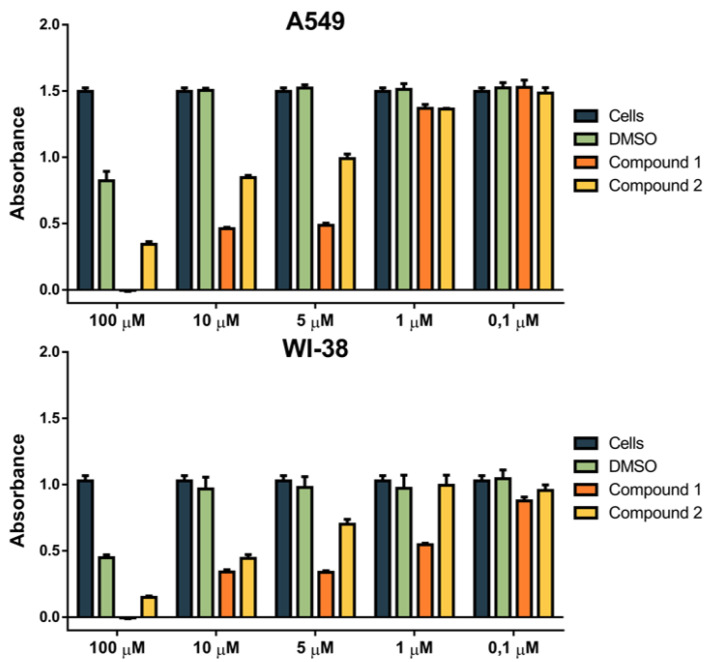
Cytotoxic assay of compounds **1** and **2** performed on A549 and WI-38 cell line. Measured absorbance (λ = 600 nm) directly correlates with cell survival. Cells with added DMSO in corresponding concentration represent control (note toxicity of DMSO at 100 µM but negligible effects at lower conc.). Data are presented as mean ± SD made in three replicates. The representative data of two independent experiments that yielded similar results are shown.

**MTT assay**. The studied compounds were screened by the MTT assay for anti-proliferative activity against human lung carcinoma (A549) and normal lung (WI38) cell line (Figure 4). Results showed moderate cytotoxic effects on the A549 cell line (IC50 of compounds **1** and **2** is approximately 8 and 10 μM, respectively), and somewhat weaker activity on the WI-38 cell line (IC50 of 15 μM for **1** and 10 μM for **2**). The series of parent cyanine-amino acids showed similar cytotoxicity [19]; thus, the addition of GCP did not have a significant influence on the anti-proliferative activity of conjugates.

**Figure 5:**
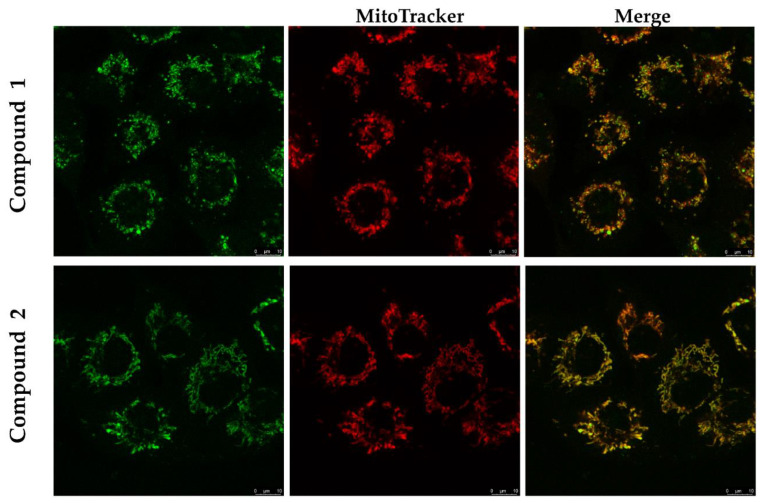
Intracellular distribution of Compounds **1** and **2** compared to MitoTracker. Confocal microscopy of live A549 cells taken on Leica SP8 X confocal microscope, stained with 10 μM of compounds **1** and **2** (λ_exc_ = 505 nm, λ_em_ = 550 nm); **RIGHT**: overlay with the MitoTracker Deep Red (λ_exc_ = 644 nm, λ_em_ = 665 nm).

**Confocal Microscopy**. All tested compounds penetrated the cell membrane of the A549 cell line within a 90 min incubation at 37 °C, 5% CO_2_. Both conjugates, **1** and **2**, being non-fluorescent in a free state, revealed strong green emission upon accumulating in mitochondria, as proven by co-localization with MitoTracker Deep Red (Figure 5).

In order to quantify the co-localization, we used Pearson’s correlation coefficient (PCC) [34], corrected for noise by Replicate-based noise correction correlation (RBNCC) [35]. The results indicated excellent co-localization of the studied dyes with the commercial mitochondrial tracker (PCC 0.91 ± 0.02).

Obtained results pointed out that **1** and **2** specifically accumulate in mitochondria, same as parent cyanine-amino acids [19], thus showing that conjugation with GCP did not influence intracellular uptake and targeting. It should be stressed that the original fluorophores TO and YO, as well as their derivatives (TOTO, YOYO, and their analogs), are typical DNA intercalators, and if able to enter cells, they act as nuclei fluorescence stains [7]. Further, novel conjugates **1** and **2** are slightly more toxic than parent cyanine-amino acids [19] and thus possess somewhat increased theragnostic ability—to fluorescently mark mitochondria and impair cellular function.

## 3. Conclusions

The here presented study showed that the linker tethering position on cyanine moiety has a strong impact on the intrinsic properties of cyanine-amino acid-guanidiniocarbonyl-pyrrole conjugates in water. Namely, a derivative with a positive charge more exposed to the solvent (Scheme 1, **2**) showed significantly stronger aggregation propensity with respect to the analog with a more centered positive charge (Scheme 1, **1**). Also, **1** and **2** showed significantly different fluorimetric and circular dichroism (induced CD) sensing of various ds-DNA and ds-RNA. The analog with a more centered positive charge (Scheme 1, **1**) showed pronounced fluorimetric and CD selectivity toward AU-RNA, while the positive charge of **2** was more exposed to the solvent almost completely abolished fluorimetric selectivity. However, as a result of more pronounced aggregation ability, only **2** showed measurable induced (I)CD bands, yielding different ICD band patterns for AT(U)-polynucleotides with respect to GC-containing-polynucleotides.

Interactions of both studied compounds with DNA/RNA were, to some extent, controllable by pH due to GCP protonation, yielding somewhat stronger affinity and thermal stabilization effects at pH 5. However, pH control did not result in pronounced differences like noticed for pyrene analogs PE1 (at pH 5, an opposite emission change of GC- or AT-DNA, respectively) [15], as well as for other pyrene-GCP analogs [13,14]. The reason is cyanine fluorophore being cationic itself, and thus not so dependent in binding to DNA/RNA on the additional positive charge of GCP present only at pH 5.

Preliminary screening on human tumor and normal lung cell lines showed that both dyes very efficiently enter living cells and specifically accumulate in mitochondria. Although spectrophotometric results showed that studied compounds bind equally efficiently to all ds-DNA or ds-RNA, intracellular localization is quite specific for mitochondria. Since cyanine dye used in the construction of compounds (TO) usually localizes in the cell nucleus, it seems that amino acid residue with GCP unit changes the target, likely because of the fine equilibrium of steric, hydrophobic, and H-bonding interactions. To determine the exact set of interactions, which take place *in cellulo*, further experiments are needed, which would in detail characterize cellular uptake mechanism (likely related to some of the membrane protein transporters, binding studied molecules and transporting them into mitochondria), and then in mitochondria determining whether ds-DNA or protein is the main target.

The introduction of GCP did not have a pronouncedly different biological effect in comparison to parent cyanine-amino acid; nevertheless, the novelty is that for the first time, the GCP unit was introduced into mitochondria, thus proving that cyanine-amino acids can be used as mitochondria-targeting transporters even for rather large molecular moieties.

Thus, compounds **1** and **2** are behaving as intriguing novel mitochondria-specific theragnostic dyes. Namely, similar molecules or supramolecular constructs characterized by simultaneous highly selective intracellular fluorescence localization and biological effects (many of them cyanine analogs), are increasingly studied for so-called theragnostic (therapy and diagnostic) applications [36,37,38]. Further development of here presented lead compounds (**1**, **2**) would explore the applicability of pH (based on GCP moiety) or thermochemical control of mitochondrial function as a tool of controlling cell life cycles.

## 4. Materials and Methods

### 4.1. General Information

Solvents were distilled from appropriate drying agents shortly before use. TLC was carried out on TLC Silica gel 60 F254 Plastic sheets, and preparative thin layer (2 mm) chromatography was done on Merck 60 F254 plates (Merck KGaA, Darmstadt, Germany). NMR spectra were recorded on AV600 and AV300 MHz spectrometers (Bruker BioSpin GmbH, Rheinstetten, Germany), operated at 600.13 or 300.13 MHz for ^1^H nuclei and at 150.92 MHz or 75.46 MHz for ^13^C, using DMSO-*d*_6_ (δ_H_: 2.50 ppm, δ_C_: 39.52 ppm) or CDCl_3_ (δ_H_: 7.26 ppm, δ_C_: 77.16 ppm) as the internal standard. Mass spectrometry was performed on an Agilent 6410 Triple Quad mass spectrometer (Agilent Technologies, Santa Clara, CA, USA). High-resolution mass spectra (HRMS) were obtained using a Q-TOF2 hybrid quadrupole time-of-flight mass spectrometer (Micromass, Cary, NC, USA).

### 4.2. Synthesis

***Compound 1***

*(E)-5-bromo-3-(4-(4-(2-(5-(carbamimidoylcarbamoyl)-1H-pyrrole-2-carboxamido)-3-methoxy-3-oxopropyl)-1H-1,2,3-triazol-1-yl)butyl)-2-((1-methylquinolin-4(1H)-ylidene)methyl)benzo[d]thiazol-3-ium) was prepared previously.13*

***Compound 2***

*(E)-5-bromo-2-((1-(4-(4-(2-(5-(carbamimidoylcarbamoyl)-1H-pyrrole-2-carboxamido)-3-methoxy-3-oxopropyl)-1H-1,2,3-triazol-1-yl)butyl)quinolin-4(1H)-ylidene)methyl)-3-methylbenzo[d]thiazol-3-ium)*

The reaction mixture of Cy-amino acid and GCP in acetonitrile with HCTU and Et_3_N was left stirring at room temperature under argon for 3 days. Boc-protected product was deprotected by the addition of TFA, the solvent was evaporated, and the crude product was recrystallized from the methanol-ether mixture and washed with water to give a pure product in 60% yield as a red solid.

**^1^H NMR** (600 MHz, DMSO) δ 12.69 (s, 1H, NH), 12.46 (s, 1H, NH), 10.95 (s, 1H, NH), 8.83 (dd, *J* = 24.5, 13.1 Hz, 1H, Ar), 8.65 (d, *J* = 38.3 Hz, 1H, Ar), 8.16 (d, *J* = 12.2 Hz, 2H, Ar), 8.06–8.00 (m, 3H, Ar + NH), 7.98–7.91 (m, 2H, Ar), 7.76 (d, *J* = 7.3 Hz, 2H, Ar), 7.67 (d, *J* = 8.2 Hz, 1H, Ar), 7.56 (d, *J* = 5.0 Hz, 1H, Ar), 7.38 (d, *J* = 15.1 Hz, 1H, Ar), 7.03 (t, *J* = 25.5 Hz, 1H, Ar), 6.93–6.82 (m, 2H, Ar), 6.70 (s, 1H, CH), 4.63 (dd, *J* = 57.4, 14.8 Hz, 2H, CH2), 4.43–4.30 (m, 1H, CH), 4.00 (s, 3H, CH3), 3.84 (d, *J* = 22.3 Hz, 2H, CH2), 3.60 (s, 3H, CH3), 2.97–2.86 (m, 2H, CH2), 1.97–1.69 (m, 4H, 2XCH2) ppm.^13^C NMR (151 MHz, DMSO) δ 162.81, 160.09, 154.96, 149.00, 144.84, 142.34, 142.07, 137.15, 133.61, 127.54, 126.84, 125.85, 125.00, 124.30, 123.21, 121.52, 121.06, 116.70, 115.57, 108.36, 88.38, 83.51, 54.22, 52.43, 34.30, 33.95, 26.11 ppm. HRMS (MALDI-TOF/TOF): *m*/*z* calcd for C_35_H_37_BrN_10_O_4_S^2+^ ([M − H]^+^) 773,1980, found 773,1982.

### 4.3. Study of DNA/RNA Interactions

All measurements were performed in aqueous buffer solution (pH = 7.0 or pH 5.0, *I* = 0.05 M, sodium cacodylate/HCl buffer). The UV-Vis spectra were recorded on a Varian Cary 100 Bio spectrometer, fluorescence spectra were recorded on a Varian Cary Eclipse fluorimeter, and CD spectra were recorded on JASCO J815 spectropolarimeter at 25.0 °C using appropriate quartz cuvettes (path length: 1 cm).

Polynucleotides were purchased as noted: poly dAdT–poly dAdT, poly A–poly U, poly A, poly G, poly C, poly U (Sigma), *calf thymus* (ct)-DNA (Aldrich) and dissolved in sodium cacodylate buffer, *I* = 0.05 M, pH = 7.0. The ct-DNA was additionally sonicated and filtered through a 0.45 mm filter to obtain mostly short (ca. 100 base pairs) rod-like B-helical DNA fragments [39]. Polynucleotide concentration was determined spectroscopically [40] as the concentration of phosphates (corresponds to *c*(nucleobase)).

Circular dichroism (CD) spectra were recorded on JASCO J-815 spectropolarimeter at room temperature using 1 cm path quartz cuvettes with a scanning speed of 200 nm/min (an average of three accumulations). A buffer background was subtracted from each spectrum. CD experiments were performed by adding portions of the compound stock solution into the solution of the polynucleotide (*c* = 2 × 10^–5^ M).

Thermal melting curves for ds-DNA, ds-RNA, and their complexes with studied compounds were determined as previously described [41] by following the absorption change at 260 nm as a function of temperature. The absorbance of the ligands was subtracted from every curve, and the absorbance scale was normalized. *T_m_* values are the midpoints of the transition curves determined from the maximum of the first derivative and checked graphically by the tangent method [15]. The Δ*T_m_* values were calculated subtracting *T_m_* of the free nucleic acid from *T_m_* of the complex. Every Δ*T_m_* value here reported was the average of at least two measurements. The error in Δ*T_m_* is ± 0.5 °C.

### 4.4. Biological Evaluation of 1 and 2 on Human Cell Lines

**Study model.** Experiments have been performed using two human cell lines, epithelial human lung adenocarcinoma A549 (ATCC® CCL-185™) and human normal lung fibroblast WI-38 (ATCC® CCL-75™). Both cell lines adhere to plastic and glass surface and are maintained in the culture under same conditions. Cells were grown in Dulbecco Modified Eagle′s Medium (DMEM, Sigma Aldrich, St Louis, MO, USA) supplemented with 10% of fetal bovine serum (FBS, Sigma Aldrich, St Louis, MO, USA) at 37 °C and 5% CO_2_ in a humified atmosphere. Cells were passaged twice per week in order to retain maximum confluency of 70–80%. Cells exhibiting normal morphology without any contamination signs, were kept in culture and used in all further experiments. Two biological replicas have been performed for all experiments.

**Cytotoxicity assay—MTT.** Studied compounds **1** and **2** were dissolved in an appropriate volume of dimethyl sulfoxide solution (DMSO, Gram-Mol, Zagreb, Croatia) under sterile conditions, in order to obtain the stock of 10 mM solution. Solutions were kept in the dark and stored at −20 °C in order to prevent degradation. Prior to each assay, a new fresh working solution has been prepared from the stock solution. For testing the cytotoxic effects of each chemical compound on A549 and WI-38 cells, stock solutions have been diluted with DMEM (10% FBS) to get a range of concentrations (100, 10, 5, 1, and 0.1 μM) which have been tested using a MTT test. Cells were seeded on a 96 well plate at a concentration of 7 × 10^3^ cells/well in 100 μL of DMEM (10% FBS) and left in the incubator overnight (37 °C, 5% CO_2_). The next day, 100 μL of the working solution was added to the wells in that manner that the final concentration of tested compounds was obtained in the total volume of 200 μL/well. All conditions were tested in triplicates. Cells treated with the same dilutions of DMSO represented control, while cells treated only with DMEM (10% FBS) represented negative control. The plate was then incubated for the next 72 h (37 °C, 5% CO_2_). After the incubation, the medium was removed, and the 40 μL of MTT solution was added to each well (treated wells, control wells containing DMSO, cells grown only in medium, and the empty well, served as blank). The plate was incubated in the cell incubator for 3 h, allowing the formazan crystals to form. After 3 h, 170 μL of DMSO was added in each well and put on a shaker for 20 minutes, allowing crystals to dissolve. The absorbance of the MTT-formazan product was measured with a microplate reader at 600 nm. Absorbance value directly correlates with a cell survival.

**Co-localization Assay**. Live imaging of the cells treated with compounds was performed on the A549 cell line. Cells were seeded in Ibidi imaging cell chambers (Ibidi®, Germany) in 500 μL of medium, with the concentration of 5 × 10^4^ cells/well, and left in the cell incubator for 48 h (37 °C, 5% CO_2_). After two days, cells were treated with a 10 μM solution of each compound and left in the cell incubator for 90 min to allow the compound to enter the cells. After the incubation, the medium was changed, and 500 μL of 100 nM MitoTracker Deep Red solution (Invitrogen, Molecular Probes) was added to the chambers. Cells were incubated for 20 min (37 °C, 5% CO_2_), allowing MitoTracker to enter the cells. After incubation, the medium was replaced with 500 μL of fresh medium. Co-localization of compounds (λ_exc_ = 505 nm, λ_em_ = 550 nm) and mitochondria (MitoTracker λ_exc_ = 644 nm, λ_em_ = 665 nm) was then visualized and confirmed using Leica SP8 X confocal microscope (Leica Microsystems, Germany).

## Figures and Tables

**Table 1:** *ΔT_m_*-Values ^a^ (°C) for different ratios ^b^ ***r*** of **1** and **2** added to polynucleotide.

	^b^ r	ct-DNA	p(dAdT)_2_	pApU
**1, pH 7.0**	0.1	2.0	0.6	1.0
	0.2	2.0	0.3	1.0
**1, pH 5.0**	0.1	2.0	1.3	^c^ 7.0; 2.0
	0.2	2.0	2.6	^c^ 6.0; 1.0
**2, pH 7.0**	0.1	1.1	1.0	2.0
	0.2	1.7	1.1	3.0
**2, pH 5.0**	0.1	2.0	1.0	^c^ 5.0; 2.0
	0.2	6.0	1.1	^c^ 8.0; 1.0

^a^*ΔT_m_*-Value error ± 0.5 °C; ^b^ r = (compound)/(polynucleotide); ^c^ Biphasic transitions: the first transition at *Tm* 28.5 °C is attributed to denaturation of polyA–polyU and the second transition at *Tm* 80.1 °C is attributed to denaturation of polyAH^+^–polyAH^+^ since poly A at pH 5 is mostly protonated and forms ds-polynucleotide [25,26,27].

**Table 2:** Binding constants and spectroscopic properties of complexes (^a^log *K_s_*/^b^Int) of conjugates **1** and **2** with ds-polynucleotides calculated by processing fluorimetric titrations (*c* = 5 × 10^−7^ M), at pH = 7.0 and pH = 5.0, sodium cacodylate buffer, *I* = 0.05 M.

	ctDNA	p(dAdT)_2_	p(dGdC)_2_	pApU
**1**, pH 7.0	6.1/120	6.2/40	6.4/160	6.2/450
**1**, pH 5.0	6.2/600	6.3/610	6.4/220	6.5/600
**2**, pH 7.0	6.2/300	6.1/270	6.2/480	5.6/500
**2**, pH 5.0	6.5/870	6.5/420	6.5/760	6.0/930

^a^Processing of titration data by means of Scatchard equation [28,29] gave values of ratio n[bound dye]/[polynucleotide] = 0.1 and 0.2, for easier comparison all log Ks values were re-calculated for fixed n = 0.1. Thus, the error of binding constant value varies within the half order of magnitude, and only differences of at least one order of magnitude can be considered as significant. Correlation coefficients were > 0.99 for all calculated Ks; ^b^*Int*—fluorescence intensity of dye/polynucleotide complex calculated by Scatchard equation, taking into account that free dyes were non-emissive.

## Supplementary Materials

Supplementary Materials are available online, structural properties of studied DNA and RNA; physico-chemical properties of studied compounds aqueous solutions, additional experimental data (fluorimetric and CD titrations, thermal denaturation experiments) of interactions with double-stranded DNA/RNA.
