# Supplementary material for: Fluorimetric and CD Recognition between Various ds-DNA/RNA Depends on a Cyanine Connectivity in Cyanine-guanidiniocarbonyl-pyrrole Conjugate
Source: Molecules. 2020 Sep 29;25(19):4470. doi: 10.3390/molecules25194470 (PMC7583847; doi:10.3390/molecules25194470)
Supplement: Supplementary file 1 [file molecules-25-04470-s001.pdf]

Supporting information:

Fluorimetric and CD recognition between various ds-DNA/RNA depends on a cyanine connectivity in cyanine-guanidiniocarbonyl-pyrrole conjugate

Tamara Šmidlehner,[1,4] Marta Košćak,[1] Ksenija Božinović,[2] Dragomira Majhen,[2] Carsten Schmuck, †[3] and Ivo Piantanida[1,*]

[1] Division of Organic Chemistry and Biochemistry, Ruđer Bošković Institute, Bijenička Cesta 54, 10000 Zagreb, Croatia; marta.koscak@irb.hr (M.K.), pianta@irb.hr (I.P.).

[2] Division of Molecular Biology, Ruđer Bošković Institute, Bijenička cesta 54, 10 000 Zagreb, Croatia. ksenija.bozinovic@irb.hr (K.B.), dragomira.majhen@irb.hr (D.M.)

[3] University of Duisburg-Essen, Institute of Organic Chemistry, Essen, Germany.

[4] Present address: National Institute of Chemistry, Hajdrihova 19, POBox 660, SI-1001 Ljubljana, Slovenia, tamara.smidlehner@ki.si (T.Š.).

* Correspondence: pianta@irb.hr (I.P.); Tel.: +385-1-4571-326

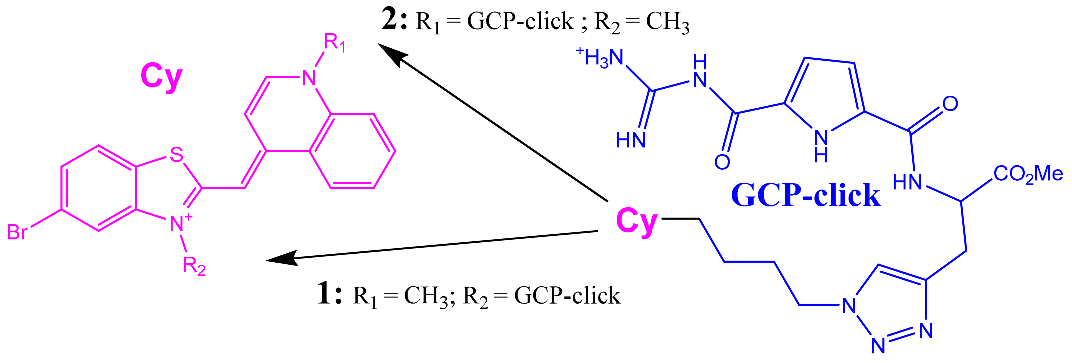

1. **Structural properties of studied DNA and RNA**
2. **Physico-chemical properties of aqueous solutions**
3. **Study of interactions of 1 and 2 with double-stranded DNA/RNA**

1. Structural properties of studied DNA and RNA

Polynucleotides were purchased as noted: poly dGdC – poly dGdC, poly dAdT – poly dAdT, poly A – poly U, *calf thymus* (ct)-DNA (Aldrich) and dissolved in sodium cacodylate buffer, I = 0.05 M, pH=7.0. The ct-DNA was additionally sonicated and filtered through a 0.45 mm filter to obtain mostly short (ca. 100 base pairs) rod-like B-helical DNA fragments [1]. The polynucleotide concentration was determined spectroscopically [2] as the concentration of phosphates (corresponds to c(nucleobase)).

Table S1. Groove widths and depths for selected nucleic acid conformation [3,4].

Structure type	Groove width [Å]		Groove depth [Å]	
	major	minor	major	minor
[a] poly rA – poly rU	3.8	10.9	13.5	2.8
[b] ct-DNA (48% of GC-pairs)	11.4	3.3	7.5	7.9
[b] poly dAdT – poly dAdT	11.2	6.3	8.5	7.5
[c] poly dGdC – poly dGdC	13.5	9.5	10.0	7.2

[a] A - helical structure

[b] B - helical structure

[c] B- helical structure with sterically blocked minor groove by amino groups of guanines

2. Physico-chemical properties of aqueous solutions

2.1. Solubility

All compounds were dissolved in water to give stock solutions of 10^{-3} M. The stock solutions where stored at -20 °C, and working aliquots kept at +25 °C. No visible precipitation or degradation was noticed over several months.

2.2. *UV/Vis and fluorescence spectra, stability*

The experiments where performed in buffer solution (sodium cacodylate buffer, *I* = 0.05 M, pH = 7.0). The absorbancies of **1** and **2** buffered solutions were proportional to their concentration within the used concentration range.

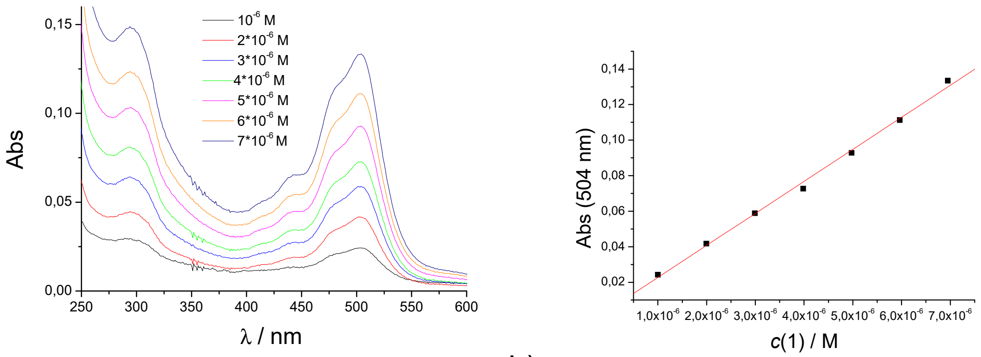

a) b)

Figure S1. a) Dependence of UV/Vis spectra on concentration of **1, b)** Dependence of Abs(504 nm) on *c* (**1**) at pH 7.0, sodium cacodylate buffer, *I* = 0.05 M.

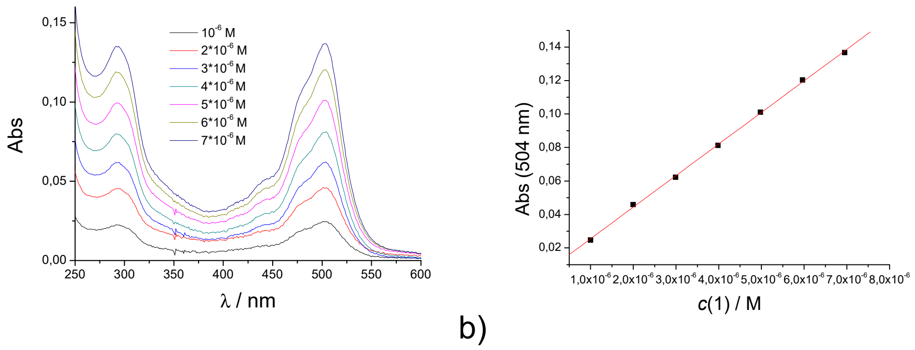

a) b)

Figure S2. a) Dependence of UV/Vis spectra on concentration of **1, b)** Dependence of Abs(504 nm) on *c* (**1**) at pH 5.0, sodium cacodylate buffer, *I* = 0.05 M.

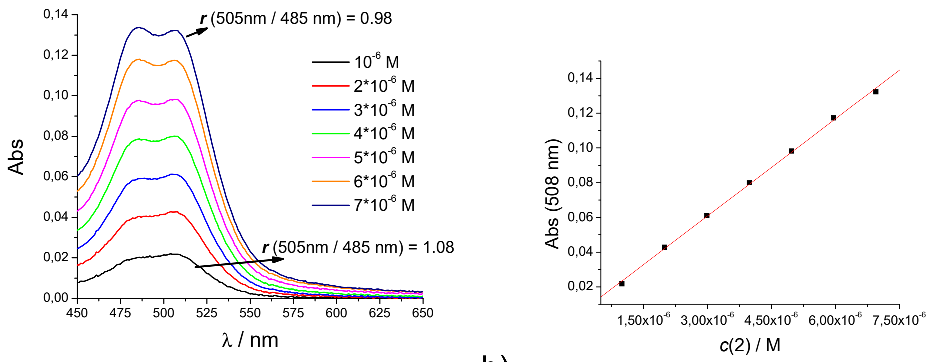

a) b)

Figure S3. a) Dependence of UV/Vis spectra on concentration of **2, b)** Dependence of Abs(504 nm) on *c* (**2**) at pH 7.0, sodium cacodylate buffer, *I* = 0.05 M.

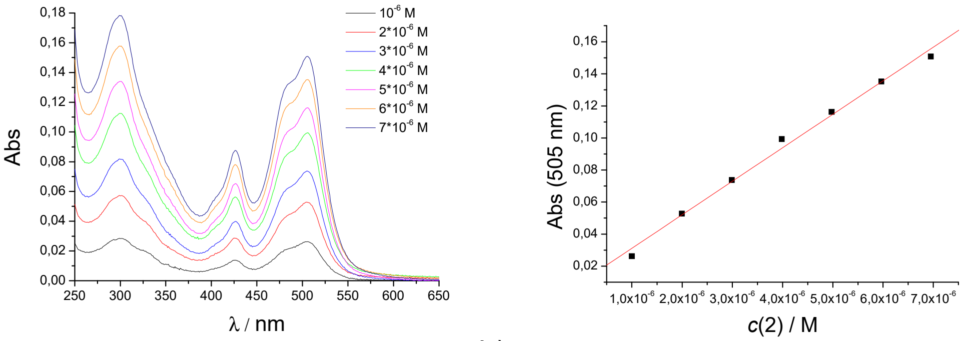

a) b)

Figure S4. a) Dependence of UV/Vis spectra on concentration of **2, b)** Dependence of Abs(504 nm) on *c* (**2**) at pH 5.0, sodium cacodylate buffer, *I* = 0.05 M.

Temperature dependence:

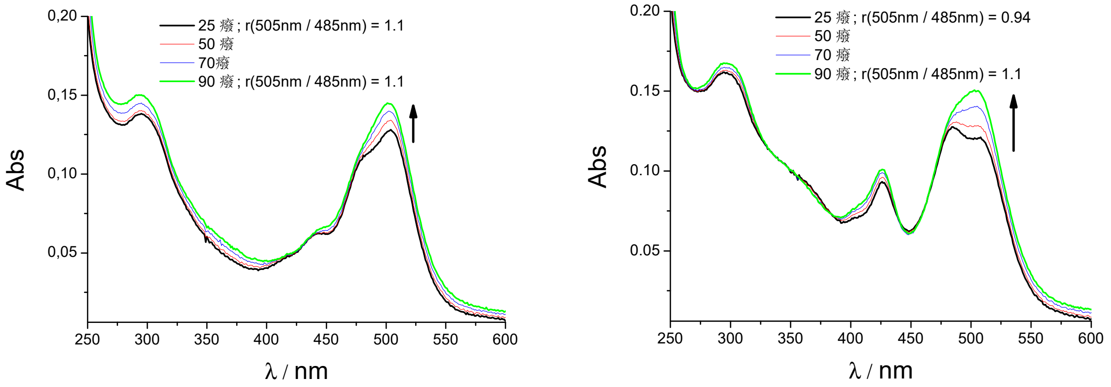

Figure S5. Temperature dependence of UV/Vis spectra (c = 1.0 × 10^{-5} M) at pH 7.0, sodium cacodylate buffer, I = 0.05 M. **LEFT: 1; RIGHT: 2**.

Table S2. Electronic absorption data of **1** and **2** determined from data on Figures S1-S4.

Compound, pH	***λ/nm***	***ε / mmol⁻¹ cm²***
1, pH 7.0	504	18018
1, pH 5.0	504	18868
2, pH 7.0	508	18629
2, pH 5.0	505	20917

3. Study of interactions with double-stranded DNA/RNA in aqueous medium

3.1 Fluorescence spectrophotometric titrations

3.1.1. Fluorescence Spectrophotometric titrations with 1

General conditions: slits 5-10; emission at 530 nm; excitation: 505 nm

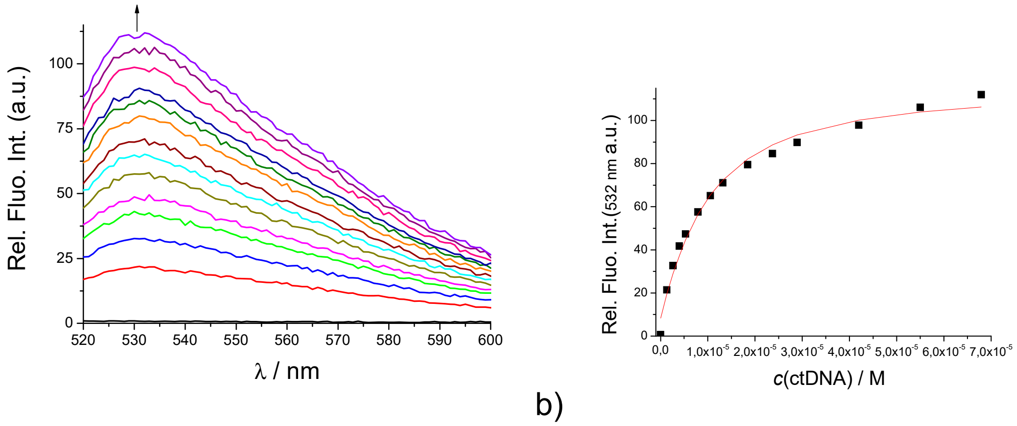

a) b)

Figure S6. a) Changes in fluorescence spectrum of **1** (c = 5.0 × 10^{-7} M, λ_{exc} = 505 nm) upon titration with ctDNA; **b)** Dependence of **1** intensity at λ_{max} = 532 nm on c(ctDNA), at pH 7.0, sodium cacodylate buffer, I = 0.05 M.

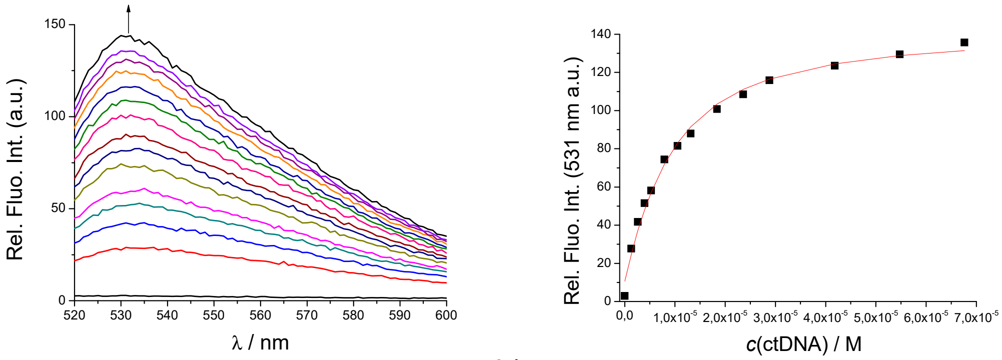

a) b)

Figure S7. a) Changes in fluorescence spectrum of **1** (c = 5 × 10^{-7} M, λ_{exc} = 505 nm) upon titration with ctDNA; **b)** Dependence of **1** intensity at λ_{max} = 531 nm on c(ctDNA), at pH 5.0, sodium cacodylate buffer, I = 0.05 M.

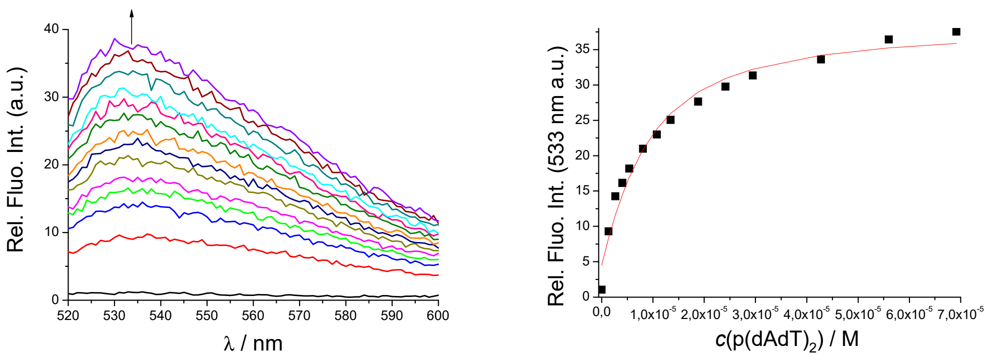

a) b)

Figure S8. a) Changes in fluorescence spectrum of **1** (c = 5 × 10^{-7} M, λ_{exc} = 505 nm) upon titration with $p(dAdT)_2$; **b)** Dependence of **1** intensity at λ_{max} = 533 nm on $c(p(dAdT)_2)$, at pH 7.0, sodium cacodylate buffer, I = 0.05 M.

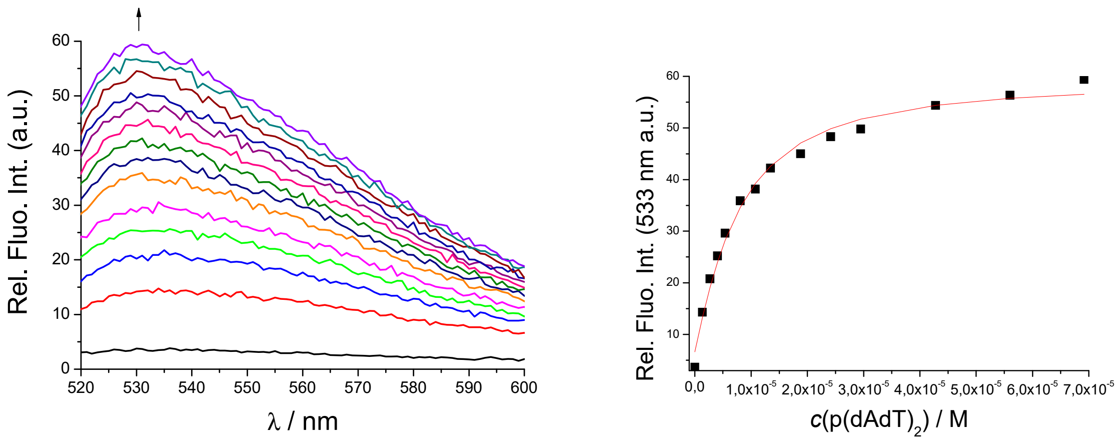

a) b)

Figure S9. a) Changes in fluorescence spectrum of **1** (c = 5 × 10^{-7} M, λ_{exc} = 505 nm) upon titration with $p(dAdT)_2$; **b)** Dependence of **1** intensity at λ_{max} = 533 nm on $c(p(dAdT)_2)$, at pH 5.0, sodium cacodylate buffer, I = 0.05 M.

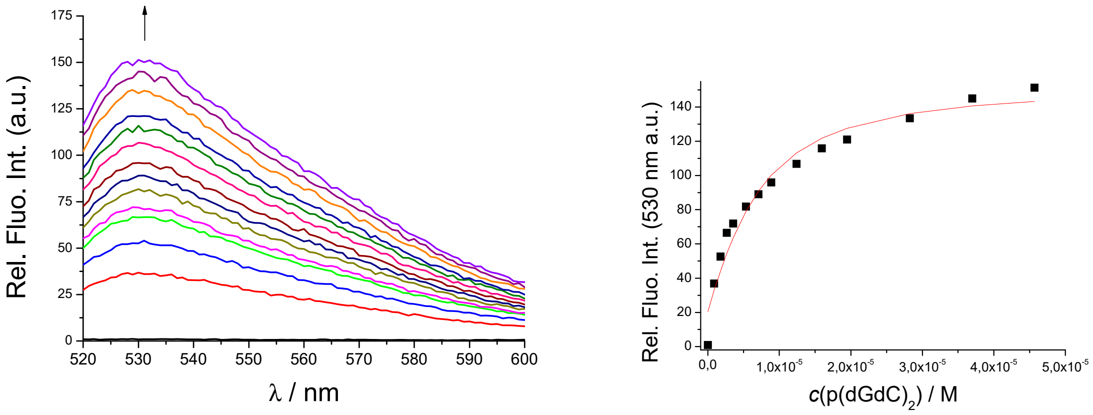

a) b)

Figure S10. a) Changes in fluorescence spectrum of **1** (c = 5 × 10^{-7} M, λ_{exc} = 505 nm) upon titration with $p(dGdC)_2$; **b)** Dependence of **1** intensity at λ_{max} = 530 nm on $c(p(dGdC)_2)$, at pH 7.0, sodium cacodylate buffer, I = 0.05 M.

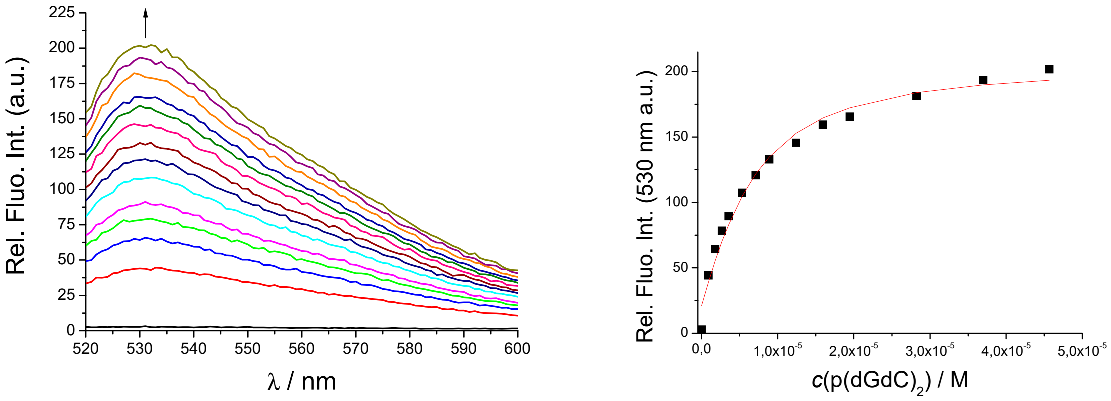

a) b)

Figure S11. a) Changes in fluorescence spectrum of **1** (c = 5 × 10^{-7} M, λ_{exc} = 505 nm) upon titration with $p(dGdC)_2$; **b)** Dependence of **1** intensity at λ_{max} = 530 nm on $c(p(dGdC)_2)$, at pH 5.0, sodium cacodylate buffer, I = 0.05 M.

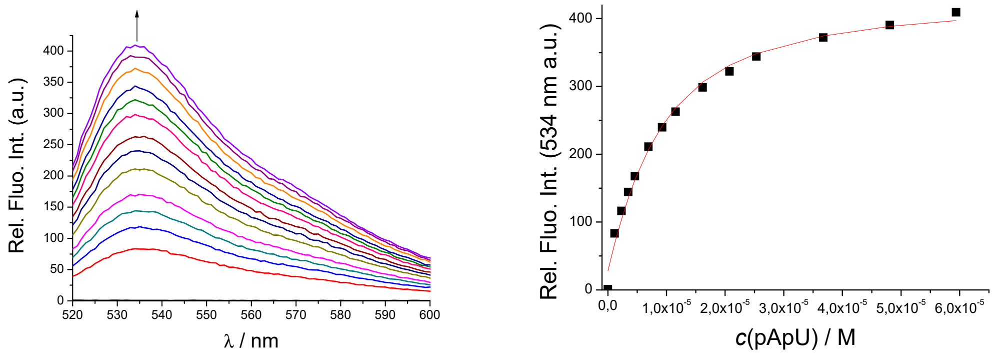

a) b)

Figure S12. a) Changes in fluorescence spectrum of **1** (c = 5 × 10^{-7} M, λ_{exc} = 505 nm) upon titration with pApU; **b)** Dependence of **1** intensity at λ_{max} = 534 nm on c(pApU), at pH 7.0, sodium cacodylate buffer, I = 0.05 M.

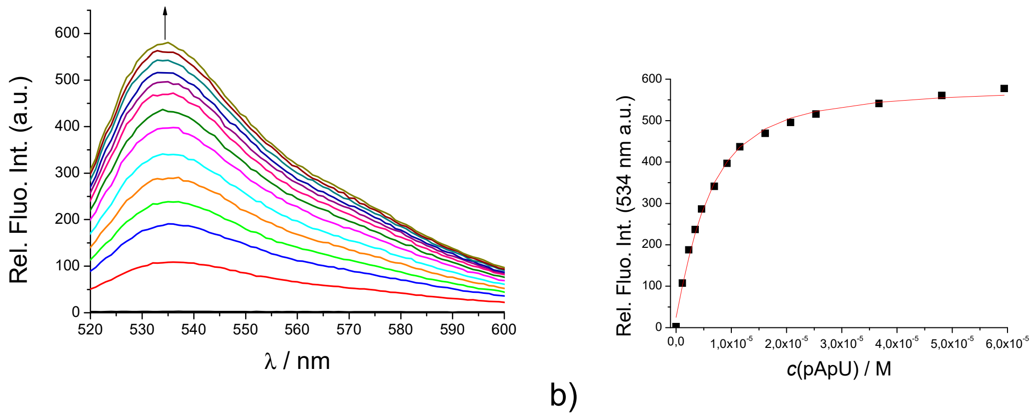

a) b)

Figure S13. a) Changes in fluorescence spectrum of **1** (c = 5 × 10^{-7} M, λ_{exc} = 505 nm) upon titration with pApU; **b)** Dependence of **1** intensity at λ_{max} = 534 nm on c(pApU), at pH 5.0, sodium cacodylate buffer, I = 0.05 M.

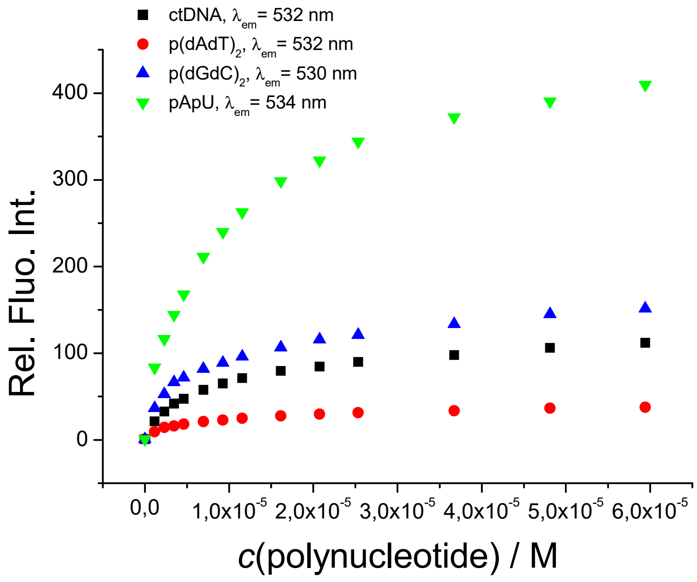

Figure S14. Changes in fluorescence of **1** (c = 5 × 10^{-7} M, λ_{exc} = 505 nm) upon addition of polynucleotides at pH 7.0.

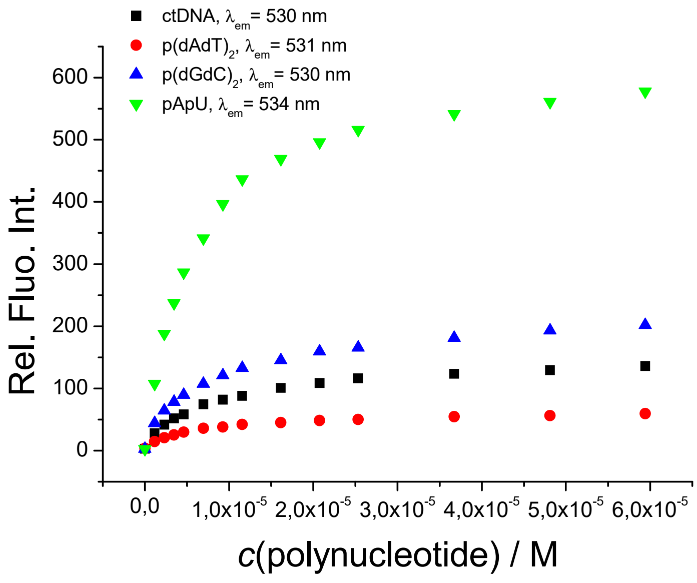

Figure S15. Changes in fluorescence of **1** (c = 5 × 10^{-7} M, λ_{exc} = 505 nm) upon addition of polynucleotides at pH 5.0.

3.1.2. Fluorescence Spectrophotometric titrations with 2

General conditions: slits 5-10; emission at 530 nm; excitation: 505 nm

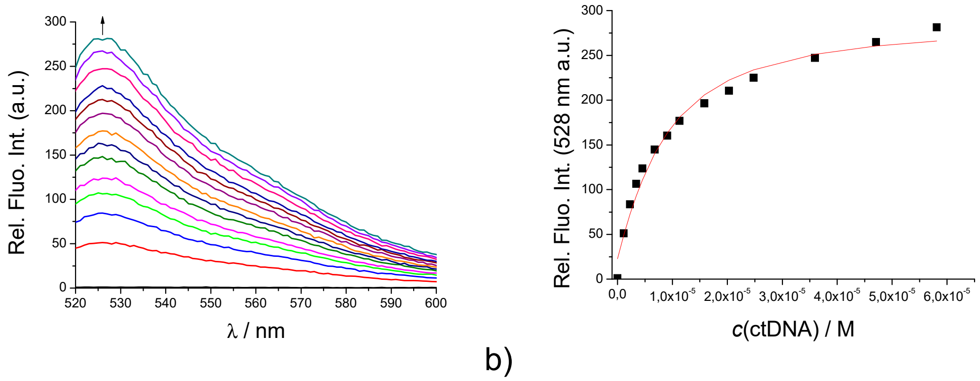

a) b)

Figure S16. a) Changes in fluorescence spectrum of **2** (c = 5.0 × 10^{-7} M, λ_{exc} = 505 nm) upon titration with ctDNA; **b)** Dependence of **2** intensity at λ_{max} = 528 nm on c(ctDNA), at pH 7.0, sodium cacodylate buffer, I = 0.05 M.

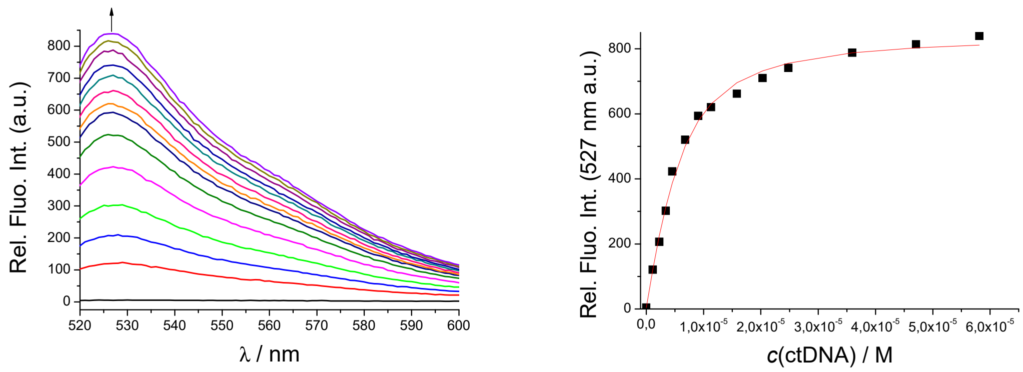

a) b)

Figure S17. a) Changes in fluorescence spectrum of **2** (c = 5.0 × 10^{-7} M, λ_{exc} = 505 nm) upon titration with ctDNA; **b)** Dependence of **2** intensity at λ_{max} = 527 nm on c(ctDNA), at pH 5.0, sodium cacodylate buffer, I = 0.05 M.

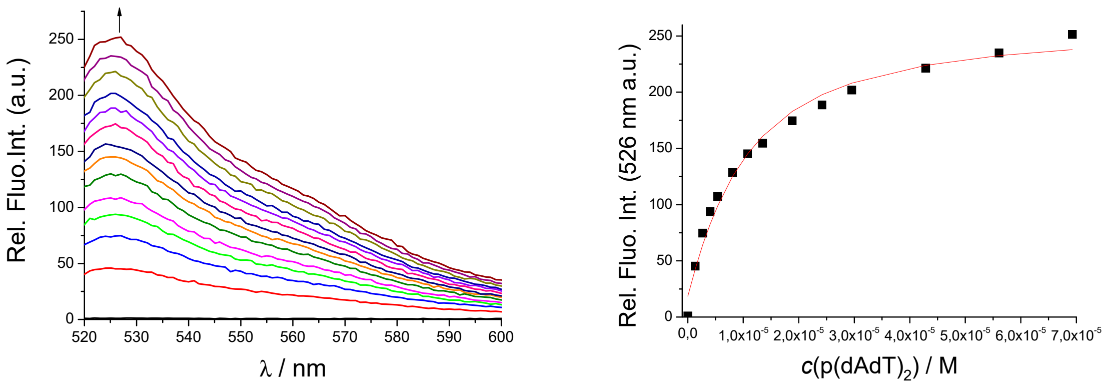

a) b)

Figure S18. a) Changes in fluorescence spectrum of **2** ($c = 5 \times 10^{-7}$ M, λ_{exc} = 505 nm) upon titration with p(dAdT)$_2$; **b)** Dependence of **2** intensity at λ_{max} = 526 nm on *c(*p(dAdT)$_2$), at pH 7.0, sodium cacodylate buffer, *I* = 0.05 M.

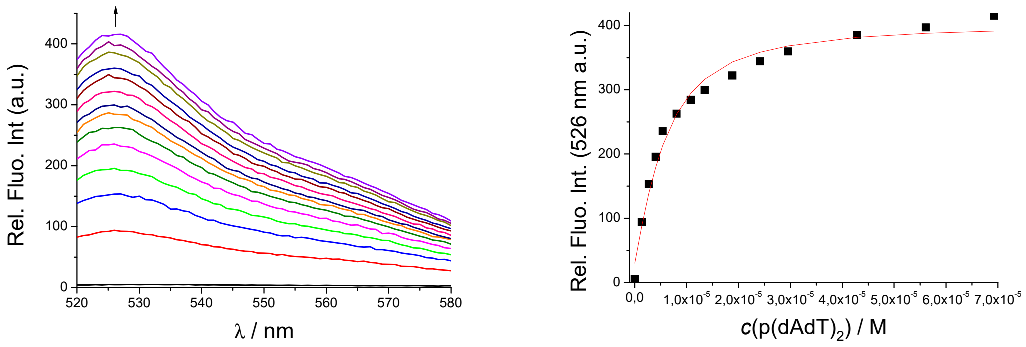

a) b)

Figure S19. a) Changes in fluorescence spectrum of **2** ($c = 5 \times 10^{-7}$ M, λ_{exc} = 505 nm) upon titration with p(dAdT)$_2$; **b)** Dependence of **2** intensity at λ_{max} = 526 nm on *c(*p(dAdT)$_2$), at pH 5.0, sodium cacodylate buffer, *I* = 0.05 M.

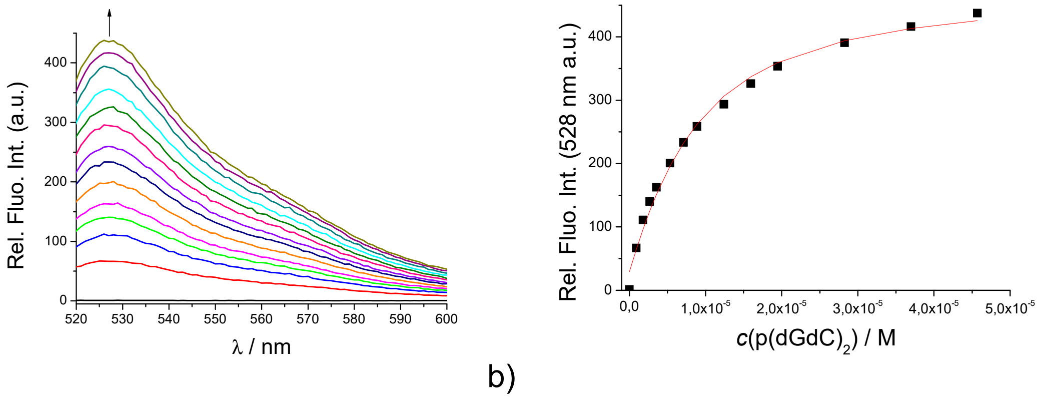

a) b)

Figure 7. a) Changes in fluorescence spectrum of **2** ($c = 5 \times 10^{-7}$ M, λ_{exc} = 505 nm) upon titration with p(dGdC)$_2$; **b)** Dependence of **2** intensity at λ_{max} = 528 nm on *c(*p(dGdC)$_2$), at pH 7.0, sodium cacodylate buffer, *I* = 0.05 M.

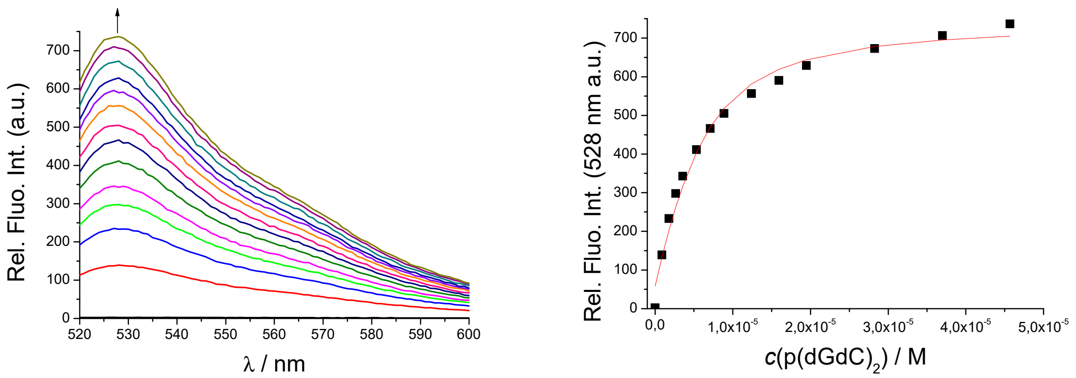

a) b)

Figure S20. a) Changes in fluorescence spectrum of **2** (c = 5 × 10^{-7} M, λ_{exc} = 505 nm) upon titration with p(dGdC)$_2$; **b)** Dependence of **2** intensity at λ_{max} = 528 nm on c(p(dGdC)$_2$), at pH 5.0, sodium cacodylate buffer, I = 0.05 M.

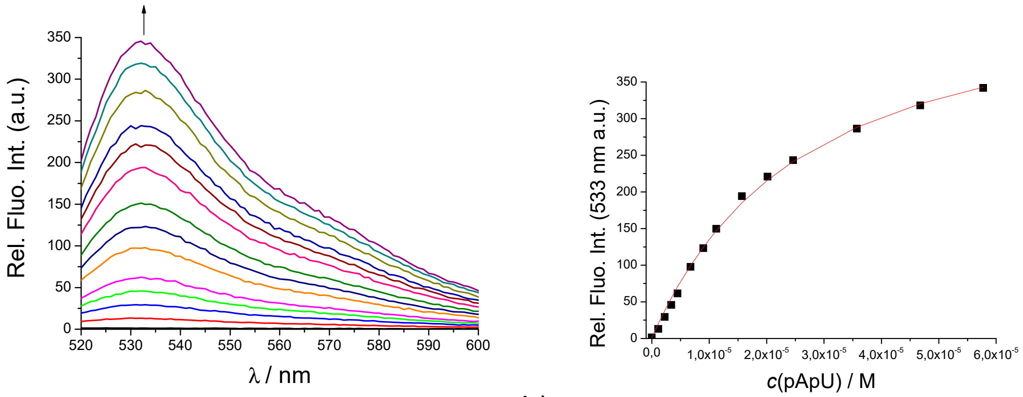

a) b)

Figure S21. a) Changes in fluorescence spectrum of **2** (c = 5 × 10^{-7} M, λ_{exc} = 505 nm) upon titration with pApU; **b)** Dependence of **2** intensity at λ_{max} = 533 nm on c(pApU), at pH 7.0, sodium cacodylate buffer, I = 0.05 M.

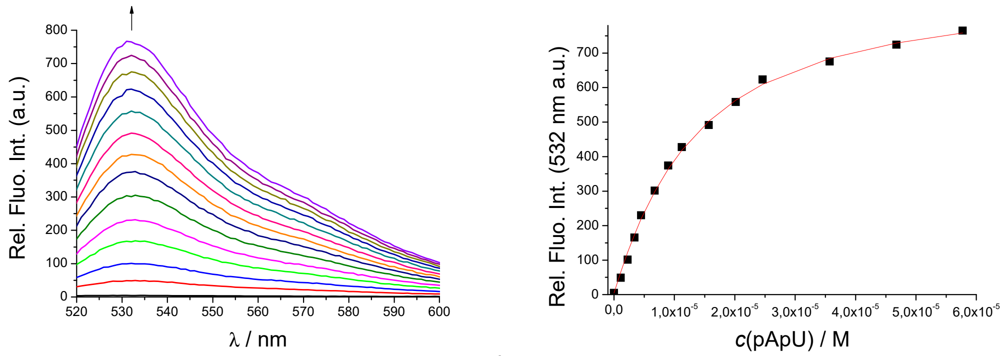

a) b)

Figure S22. a) Changes in fluorescence spectrum of **2** (c = 5 × 10^{-7} M, λ_{exc} = 505 nm) upon titration with pApU; **b)** Dependence of **2** intensity at λ_{max} = 532 nm on c(pApU), at pH 5.0, sodium cacodylate buffer, I = 0.05 M.

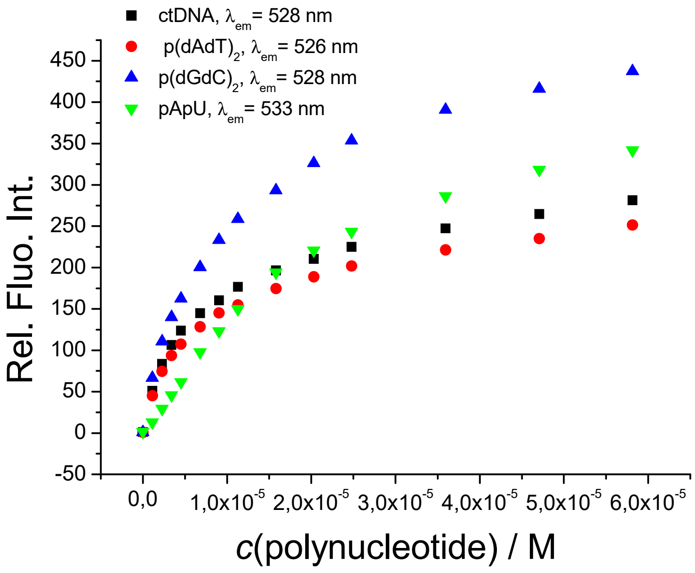

Figure S23. Changes in fluorescence of **2** (c = 5 × 10^{-7} M, λ_{exc} = 505 nm) upon addition of polynucleotides at pH 7.0.

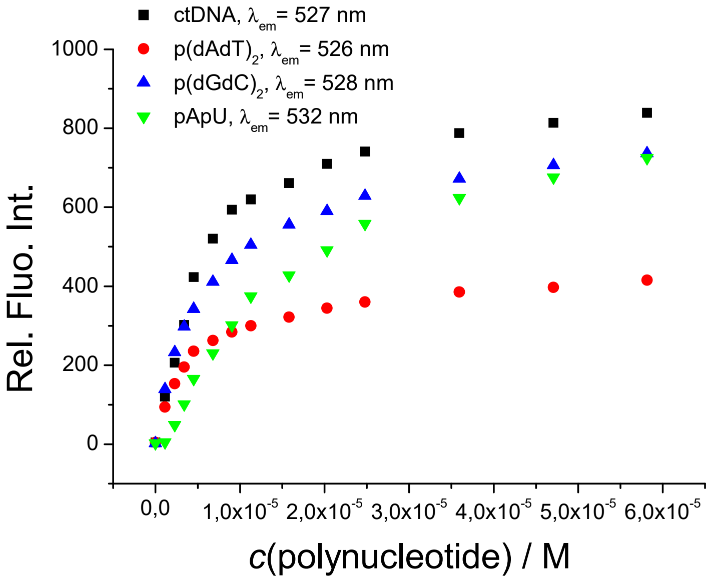

Figure S24. Changes in fluorescence of **2** (c = 5 × 10^{-7} M, λ_{exc} = 505 nm) upon addition of polynucleotides at pH 5.0.

3.2. Circular dichroism (CD) experiments

3.2.1. CD titrations with 1

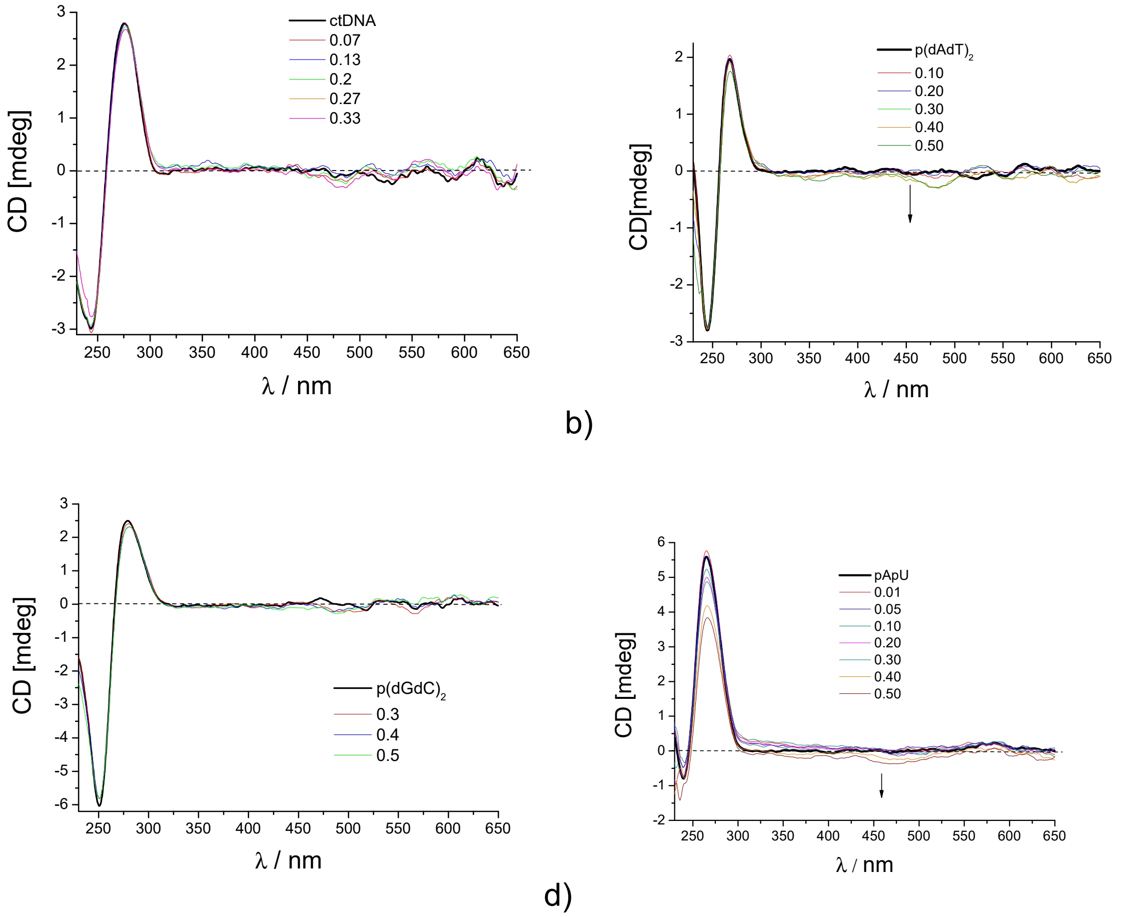

Figure S25. CD titration of a) ctDNA, b) $p(dAdT)_2$, c) $p(dGdC)_2$, d) pApU ($c = 2 \times 10^{-5}$ M) with **1** at molar ratios r = [compound] / [polynucleotide] (pH 7.0, buffer sodium cacodylate, I = 0.05 M).

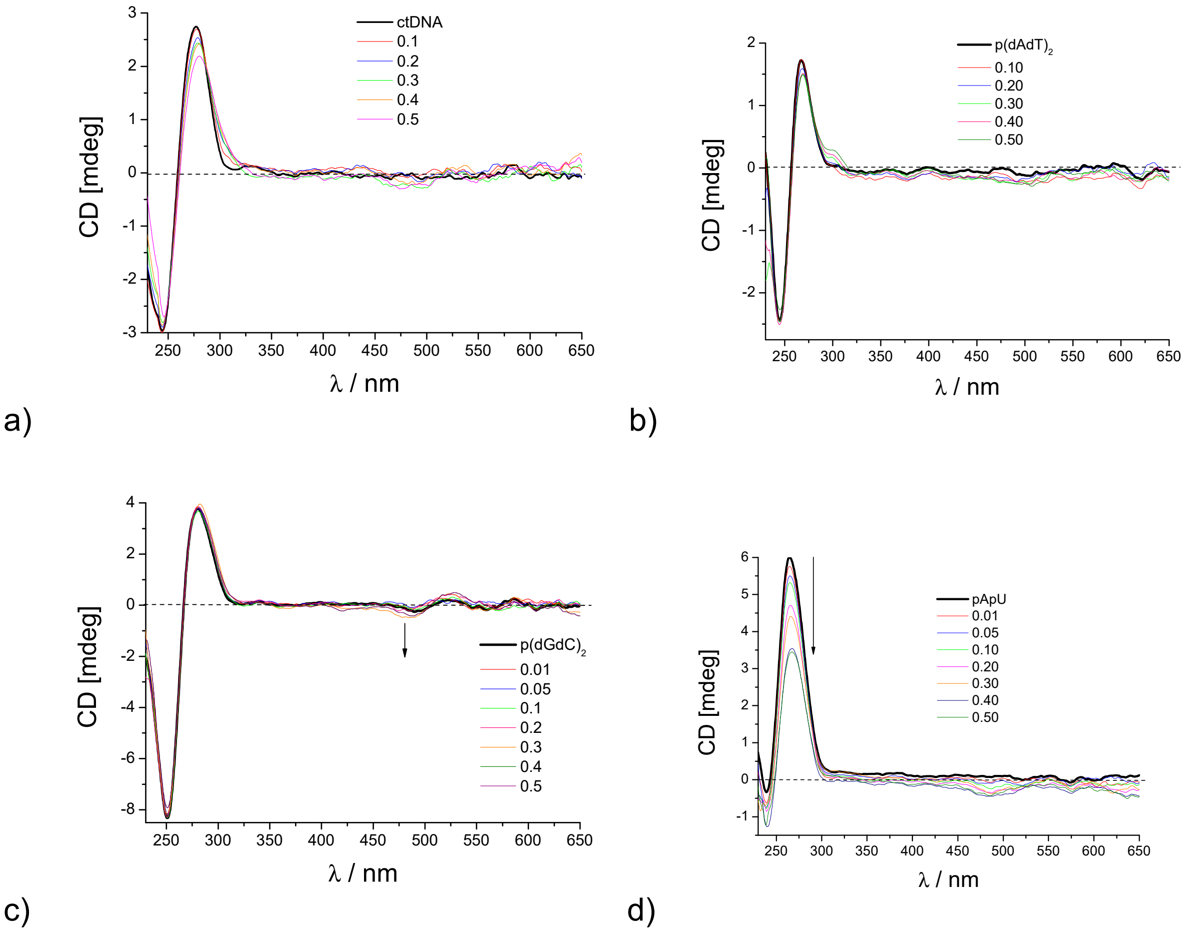

Figure S26. CD titration of a) ctDNA, b) p(dAdT)$_2$, c) p(dGdC)$_2$, d) pApU (c = 2 × 10^{-5} M) with **1** at molar ratios r = [compound] / [polynucleotide] (pH 5.0, buffer sodium cacodylate, I = 0.05 M).

3.2.2. CD titrations with 2

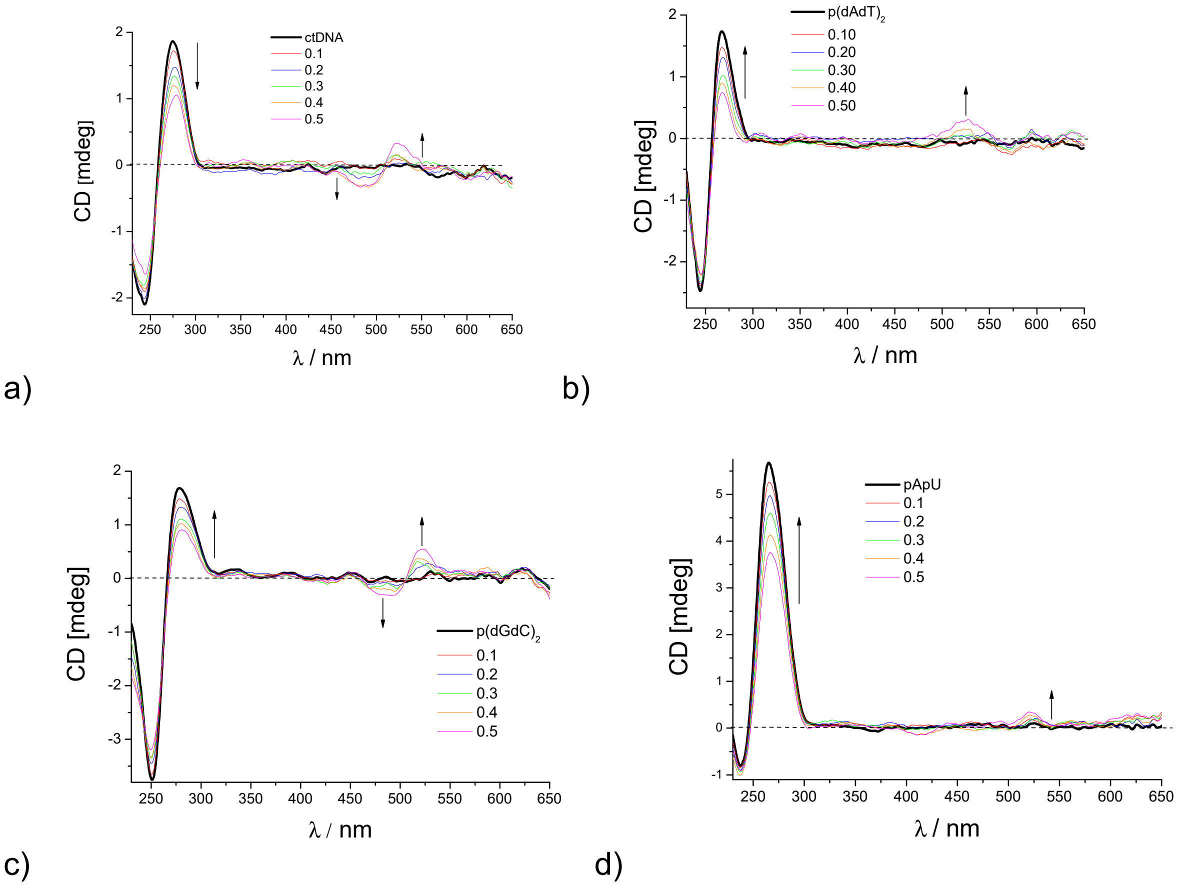

Figure S27. CD titration of a) ctDNA, b) $p(dAdT)_2$, c) $p(dGdC)_2$, d) pApU ($c = 2 \times 10^{-5}$ M) with **2** at molar ratios r = [compound] / [polynucleotide] (pH 7.0, buffer sodium cacodylate, I = 0.05 M).

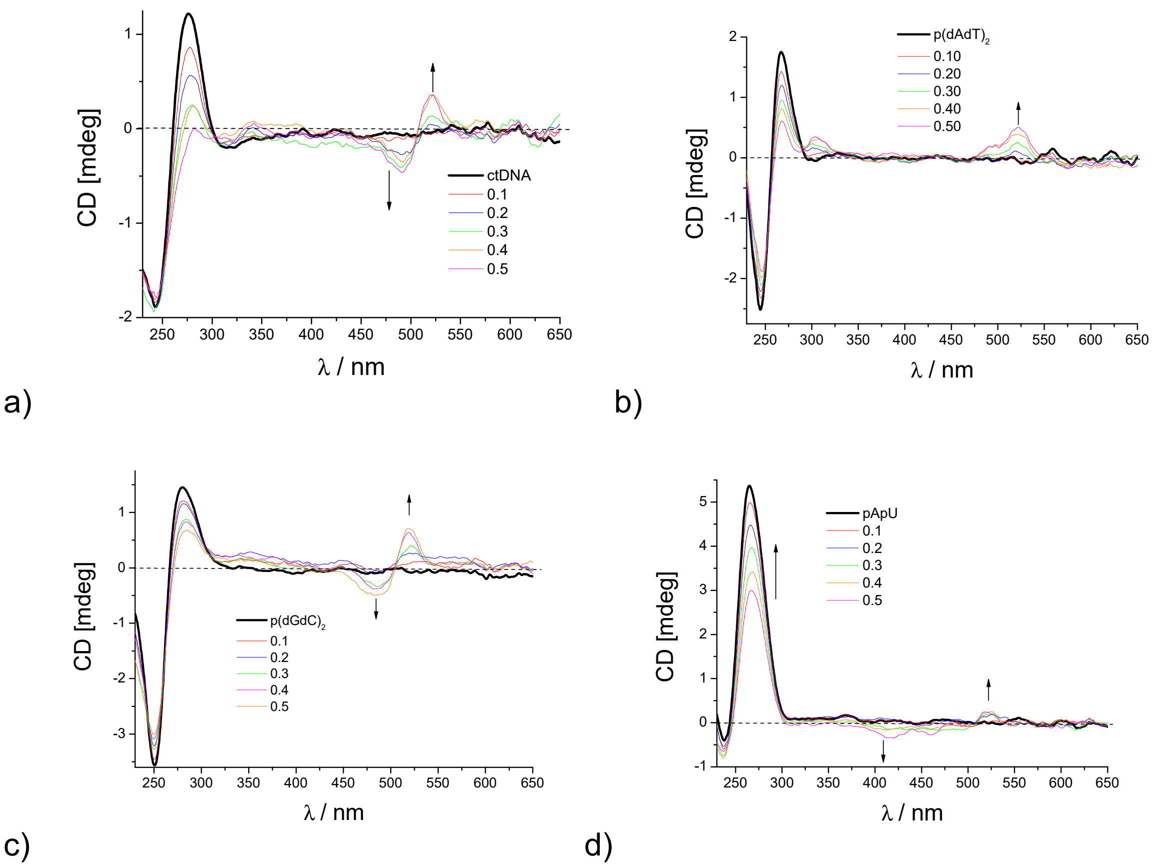

Figure S28. CD titration of a) ctDNA, b) p(dAdT)2, c) p(dGdC)2, d) pApU (c = 2 × 10^{-5} M) with **2** at molar ratios r = [compound] / [polynucleotide] (pH 5.0, buffer sodium cacodylate, I = 0.05 M).

3.3. Thermal melting experiments

3.3.1. ΔTm with 1

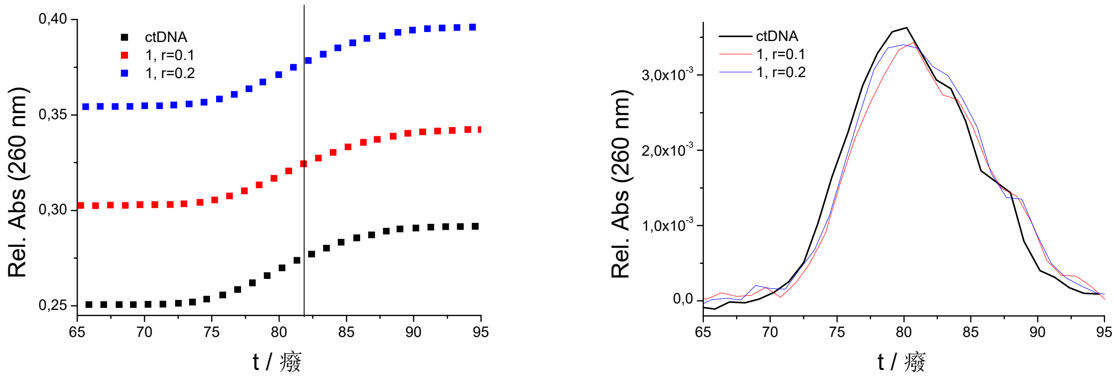

a) b)

Figure S29. a) Melting curve of ctDNA upon addition r = 0.1 and r = 0.2 ([compound/[polynucleotide]) of **1** at pH 7.0 (buffer sodium cacodylate, *I* = 0.05 M), **b)** first derivation of absorbance on temperature.

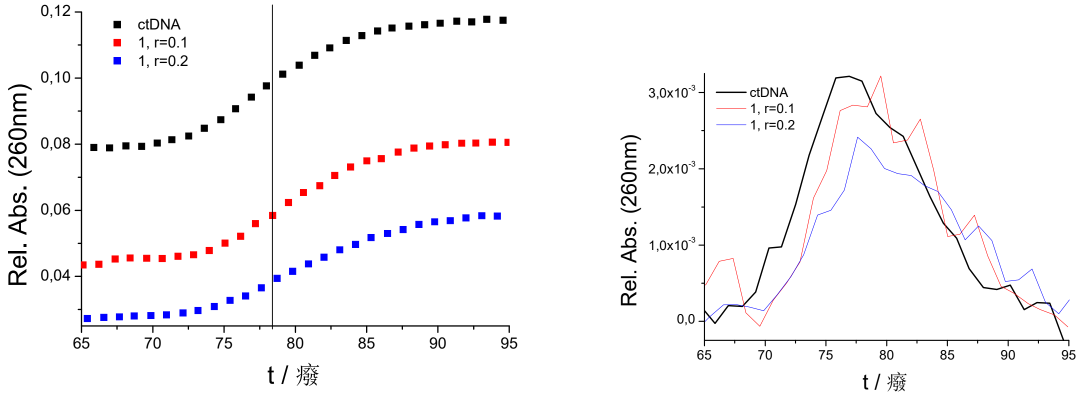

a) b)

Figure S30. a) Melting curve of ctDNA upon addition r = 0.1 and r = 0.2 ([compound/[polynucleotide]) of **1** at pH 5.0 (buffer sodium cacodylate, *I* = 0.05 M), **b)** first derivation of absorbance on temperature.

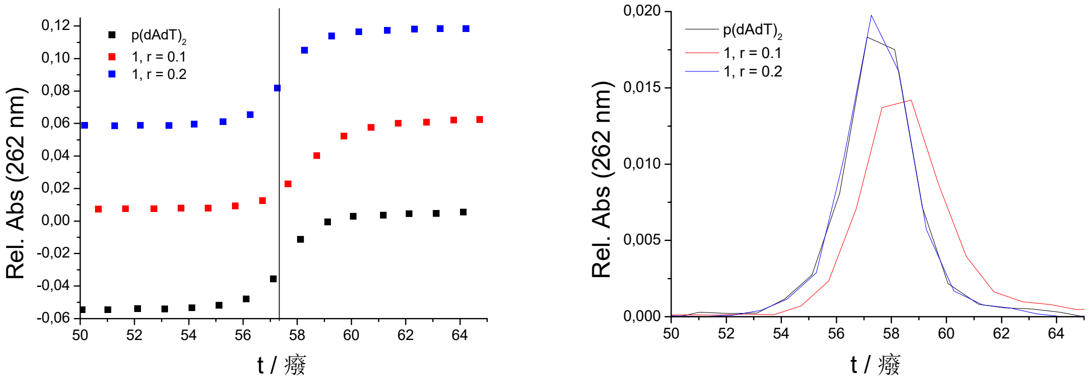

a) b)

Figure S31. a) Melting curve of p(dAdT)2 upon addition r = 0.1 and r = 0.2 ([compound/ [polynucleotide]) of **1** at pH 7.0 (buffer sodium cacodylate, *I* = 0.05 M), **b)** first derivation of absorbance on temperature

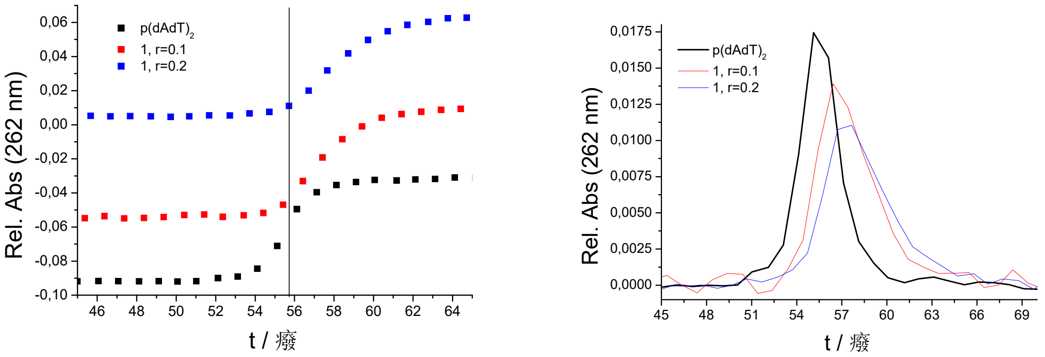

a) b)

Figure S32. a) Melting curve of p(dAdT)2 upon addition r = 0.1 and r = 0.2 ([compound/ [polynucleotide]) of **1** at pH 5.0 (buffer sodium cacodylate, *I* = 0.05 M), **b)** first derivation of absorbance on temperature

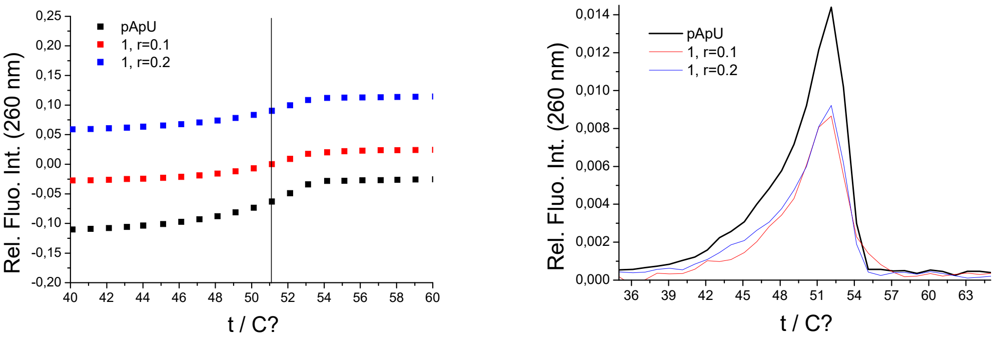

a) b)

Figure S33. a) Melting curve of pApU upon addition r = 0.1 and r = 0.2 ([compound/ [polynucleotide]) of **1** at pH 7.0 (buffer sodium cacodylate, *I* = 0.05 M), **b)** first derivation of absorbance on temperature

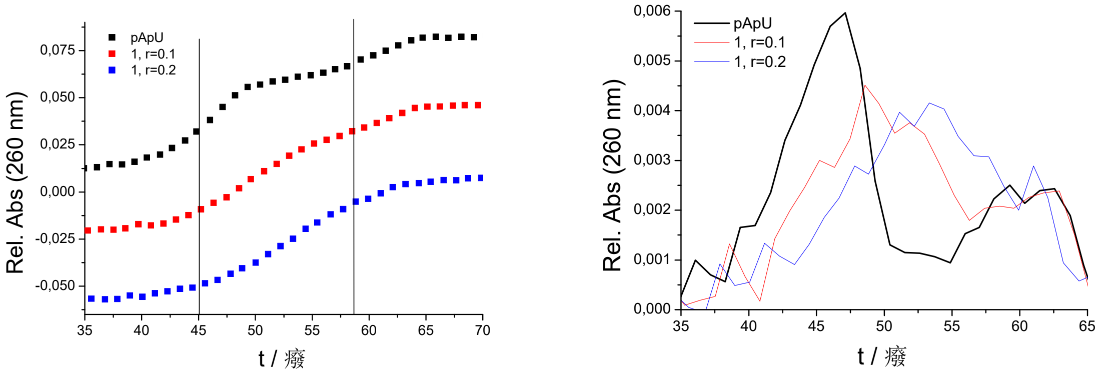

a) b)

Figure S34. a) Melting curve of pApU upon addition r = 0.1 and r = 0.2 ([compound/ [polynucleotide]) of **1** at pH 5.0 (buffer sodium cacodylate, *I* = 0.05 M), **b)** first derivation of absorbance on temperature.

3.3.2. ΔTm with 2

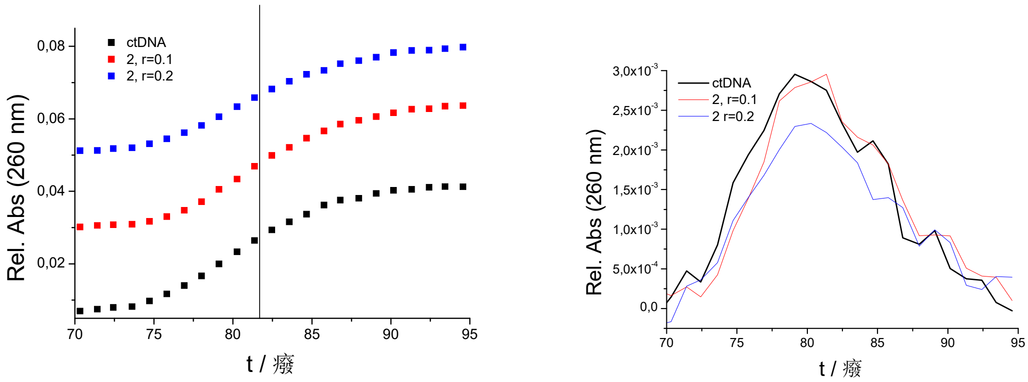

a) b)

Figure S35. a) Melting curve of ctDNA upon addition r = 0.1 and r = 0.2 ([compound/ [polynucleotide]) of **2** at pH 7.0 (buffer sodium cacodylate, *I* = 0.05 M), **b)** first derivation of absorbance on temperature

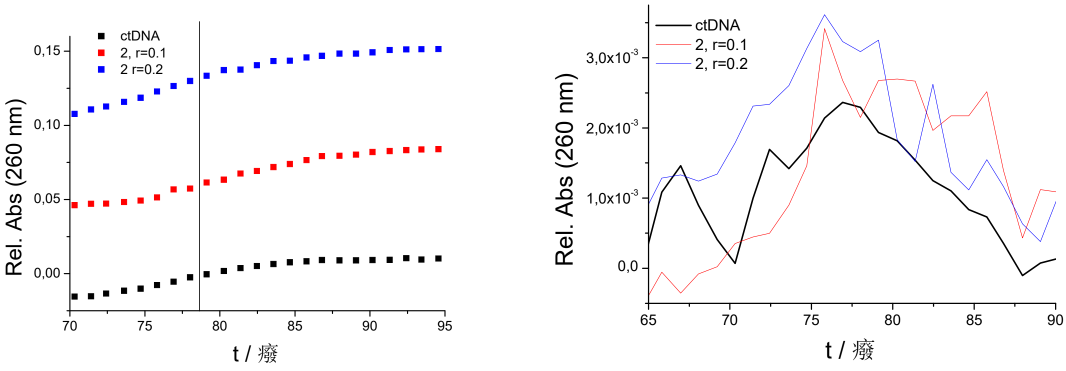

a) b)

Figure S36. a) Melting curve of ctDNA upon addition r = 0.1 and r = 0.2 ([compound/ [polynucleotide]) of **2** at pH 5.0 (buffer sodium cacodylate, *I* = 0.05 M), **b)** first derivation of absorbance on temperature

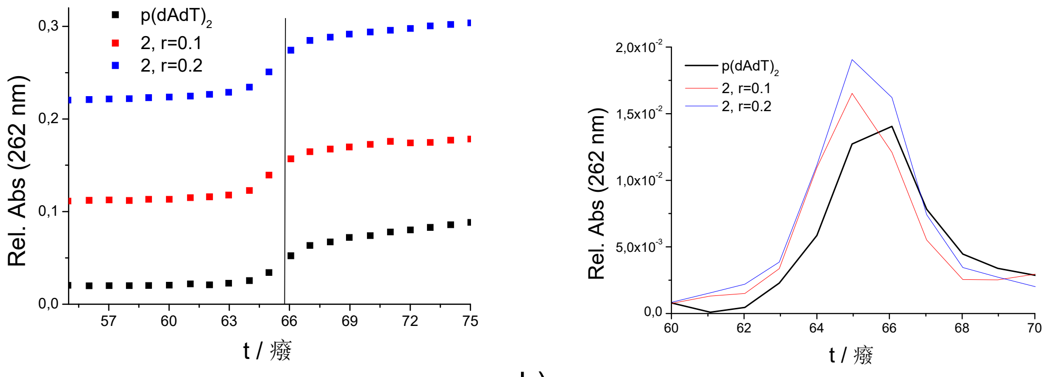

a) b)

Figure S37. a) Melting curve of p(dAdT)$_2$ upon addition r = 0.1 and r = 0.2 ([compound/ [polynucleotide]) of **2** at pH 7.0 (buffer sodium cacodylate, *I* = 0.05 M), **b)** first derivation of absorbance on temperature

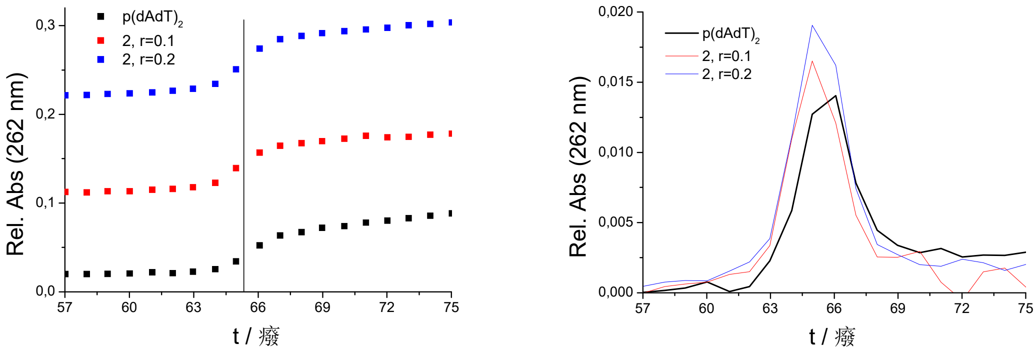

a) b)

Figure S38. a) Melting curve of p(dAdT)$_2$ upon addition r = 0.1 and r = 0.2 ([compound/ [polynucleotide]) of **2** at pH 5.0 (buffer sodium cacodylate, *I* = 0.05 M), **b)** first derivation of absorbance on temperature

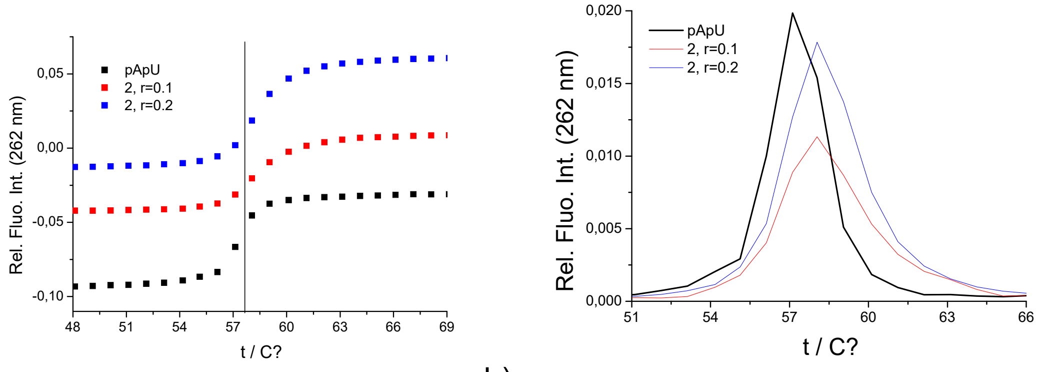

a) b)

Figure S39. a) Melting curve of pApU upon addition r = 0.1 and r = 0.2 ([compound/ [polynucleotide]) of **2** at pH 7.0 (buffer sodium cacodylate, *I* = 0.05 M), **b)** first derivation of absorbance on temperature

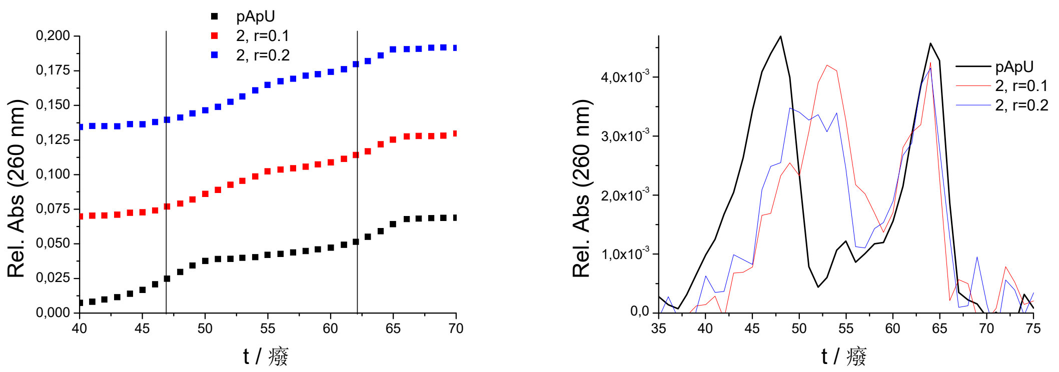

a) b)

Figure S40. a) Melting curve of pApU upon addition r = 0.1 and r = 0.2 ([compound]/[polynucleotide]) of **2** at pH 5.0 (buffer sodium cacodylate, *I* = 0.05 M), **b)** first derivation of absorbance on temperature.

---

1. Chaires, J.B.; Dattagupta, N.; Crothers, D.M. Studies on interaction of anthracycline antibiotics and deoxyribonucleic acid: Equilibrium binding studies on interaction of daunomycin with deoxyribonucleic. *Biochemistry* **1982**, *21*, 3933–3940.
2. Tumir, L.-M.; Piantanida, I.; Cindrić Juranović, I.; et al New permanently charged phenanthridinium-nucleobase conjugates. Interactions with nucleotides and polynucleotides and recognition of ds-polyAH+. *J Phys Org Chem* **2003**, *16*, 891–899, doi: 10.1002/poc.680.
3. Saenger, W. *Principles of Nucleic Acid Structure*; *Springer-Verlag* **1983,** New York.
4. Cantor, C. R.; Schimmel; P. R. Biophysical Chemistry. *WH Freeman and Co.* **1980**, *3*, 1109-1181, San Francisco.
